# The impact of allocation bias on test decisions in clinical trials with multiple endpoints using multiple testing strategies

**DOI:** 10.1186/s12874-024-02335-x

**Published:** 2024-09-30

**Authors:** Stefanie Schoenen, Nicole Heussen, Johan Verbeeck, Ralf-Dieter Hilgers

**Affiliations:** 1 Institute of Medical Statistics, RWTH Aachen University, Pauwelsstrasse 19, 52074 Aachen, Germany; 2 Medical School, Sigmund Freud Private University, Freudplatz 1, 1020 Vienna, Austria; 3 Data Science Institute, Hasselt University, Agoralaan, 3590 Diepenbeek, Belgium

**Keywords:** Allocation bias, Multiple endpoints, Šidák, All-or-none approach, Co-primary endpoints, Multiple testing, Type I error rate, Family-wise error rate, Randomization, Intersection-union test

## Abstract

**Background:**

Considering multiple endpoints in clinical trials provide a more comprehensive understanding of treatment effects and may lead to increased power or reduced sample size, which may be beneficial in rare diseases. Besides the small sample sizes, allocation bias is an issue that affects the validity of these trials. We investigate the impact of allocation bias on testing decisions in clinical trials with multiple endpoints and offer a tool for selecting an appropriate randomization procedure (RP).

**Methods:**

We derive a model for quantifying the effect of allocation bias depending on the RP in the case of two-arm parallel group trials with continuous multiple endpoints. We focus on two approaches to analyze multiple endpoints, either the Šidák procedure to show efficacy in at least one endpoint and the all-or-none procedure to show efficacy in all endpoints.

**Results:**

To evaluate the impact of allocation bias on the test decision we propose a biasing policy for multiple endpoints. The impact of allocation on the test decision is measured by the family-wise error rate of the ﻿Šidák procedure and the type I error rate of the all-or-none procedure. Using the biasing policy we derive formulas to calculate these error rates. In simulations we show that, for the ﻿Šidák procedure as well as for the all-or-none procedure, allocation bias leads to inflation of the mean family-wise error and mean type I error, respectively. The strength of this inflation is affected by the choice of the RP.

**Conclusion:**

Allocation bias should be considered during the design phase of a trial to increase validity. The developed methodology is useful for selecting an appropriate RP for a clinical trial with multiple endpoints to minimize allocation bias effects.

**Supplementary Information:**

The online version contains supplementary material available at 10.1186/s12874-024-02335-x.

## Background

According to the ICH E9, clinical trials should typically focus on a single primary endpoint, which “should be the variable capable of providing the most clinically relevant and convincing evidence directly related to the primary objective of the trial” [1]. However, in certain scenarios, considering multiple endpoints that reflect the heterogeneous clinical presentation can yield a more comprehensive understanding of treatment effects, potentially leading to increased power or reduced sample size [2]. Particularly in rare diseases it could be beneficial to include multiple endpoints due to the limited sample size [2]. Therefore, the European Medicine Agency [3] as well as the U.S. Food and Drug Association issued guidelines [4] on the use of multiple endpoints.

Regulatory guidelines propose different statistical methodologies for evaluating clinical trials with multiple endpoints, depending on their objectives and design [4]. Multiple primary endpoints are evaluated separately, so that a treatment effect on one component is sufficient to infer efficacy [5]. This approach results in an increasing number of hypotheses and thus an increased risk of erroneous conclusions, that is known as multiplicity issue [6]. A widely known approach to overcome this issue is to adapt the significance level [7]. One traditional but very conservative method is the Bonferroni procedure [6]. Therefore, we focused on the Šidák correction because it exhausts the 5% significance level exactly [8, 9]. Data-driven adjustments of the significance level, as in the Holm and Hochberg procedure [6], are less appropriate, because the impact of allocation bias should particularly be investigated in the planning phase of a clinical trial. Hence, we subsequently focus on the Šidák procedure as evaluation method for multiple primary endpoints.

In contrast to multiple primary endpoints, co-primary endpoints are used when efficacy of a treatment effect must be established for all components. In this case, the components are still analyzed simultaneously, but no adjustment of significance levels is required due to the strict decision rule [5]. One approach for analyzing a co-primary endpoint is the all-or-none procedure, commonly referred to as the intersection-union test [10]. More complex tests, such as the closed test procedure [6], can also be used to test co-primary endpoints. Since we intend to introduce a new method for evaluating the impact of allocation bias for co-primary endpoints, we initially concentrate on the less complex all-or-none procedure. Our focus in the following sections are multiple primary endpoints that are evaluated by the Šidák procedure and co-primary endpoints that are tested by the all-or-none procedure.

Bias is a widely recognized problem that affects the validity of clinical trials [1]. According to the ’Catalogue of Bias’ [11], allocation bias occurs if the structural equality of the treatment and control group is violated. If the researcher is aware of previous allocations and influences the allocation process by predicting future assignments, Berger called this third-order ’selection bias’ [12]. In the following, we will refer to this as third-order allocation bias. We aim to quantify the impact of allocation bias on the test decision, rather than ’subjective’ assessment. Therefore, we need to develop a methodology for bias assessment. The first approach for assessing allocation bias was developed by Blackwell and Hodges [13]. They devised a guessing strategy that allocates the next patient to the group with assumed fewer previous assignments. If this is the group whose superiority should be demonstrated, a patient with a better prognosis is selected. Based on this approach Proschan [14] and Langer [15] analyzed the impact of allocation bias on test decisions of the Z-test and t-test for single endpoints. The impact of allocation bias has been examined for various study designs, such as multi-arm trials [16] or survival analyses [17]. All of these bias assessment methods are provided in the R software package randomizeR [18]. However, there is a lack of studies and methods that examine the impact of allocation bias in clinical trials with multiple endpoints.

In the following, we build a model to quantify the impact of allocation bias on the test decisions based on the analysis of multiple primary endpoints or co-primary endpoints. Exemplary, we will study the Šidák and all-or-none procedure, respectively. Therefore, we proceed as follows: We start by defining the general statistical model and recap the statistical analysis methods for evaluating multiple primary endpoints and co-primary endpoints. Further, we review the randomization procedures (RPs) relevant to this paper. Then, we propose a modified allocation biasing policy for two-arm parallel group trials with continuous multiple primary and co-primary endpoints that includes endpoint-specific allocation bias effects. Based on this model, we derive formulas for the actual biased family-wise error rate (FWER) of the Šidák procedure and the actual biased type I error rate (T1E) of the all-or-none procedure. To do this we assume that the endpoint-specific hypotheses are tested by t-tests and extend Langer’s approach for single primary endpoint to the multiple case. We analyze the impact of allocation bias for different clinical settings and RPs by conducting a simulation study. We illustrate the developed methodology by means of an example. Finally, the results are summarized, potential limitations are identified and model improvements are highlighted.

## Methods

Our objective is to quantify the impact of allocation bias on the test decision for clinical trials with multiple endpoints regarding the allocation process, i.e., the RP that has to be considered. To enable the discussion some basic notations and concepts are introduced.

### Statistical model

Consider a two-arm parallel group randomized single-center clinical trial with $m\ge 2$ primary endpoints, with no interim or adaptive analysis that assesses data at a single time point. The intended allocation ratio is 1 : 1. Let $n_E$ and $n_C$ indicate the number of patients which are randomly allocated to the treatment group (E) or the control group (C), so that the total sample size is $N=n_E+n_C$. Let $\mathbf {X_j}=(X_{j,1},\dots , X_{j,m})^T$ be the multivariate normally distributed response vector of patient *j* formed by $m\ge 2$ endpoint variables where $X_{j,k}$ reflects the continuous response regarding the *k*-th endpoint. Note that the random vectors $\varvec{X_j}$, $j\in \{1,\dots ,N\}$ are independent and distributed according to

$$\begin{aligned} \mathbf {X_{j}}\sim \left\{ \begin{array}{ll} \mathcal {N}_m(\varvec{\mu _{E}},\Sigma ),& \text {if}\quad t_j=1\\ \mathcal {N}_m(\varvec{\mu _{C}},\Sigma ),& \text {if}\quad t_j=0 \end{array}\right. . \end{aligned}$$

Thereby, $t_j=1$ if patient *j* is allocated to the treatment group (E) and $t_j=0$ if patient *j* is allocated to the control group (C), $\varvec{\mu _i }= (\mu _{i,1}, \dots , \mu _{i,m})^T$, $i\in \{E, C\}$ indicates the expected response vector of the treatment or control group and $\Sigma \in \mathbb {R}^{m\times m}$ the unknown, but common covariance matrix of both groups with $\Sigma _{k,k}=Var(X_{j,k}):=\sigma _k^2$. Note that the allocation vector $\textbf{t}=(t_1,\dots ,t_N)^T$ is a realization of the random vector ${\textbf {T}}=(T_1,\dots ,T_N)^T\in \{0,1\}^N$ whose distribution is provided by the chosen RP.

To account for the impact of allocation bias on the patient responses we define the vectors $\varvec{\tau _{j}}=(\tau _{j,1},\dots ,\tau _{j,m})^T$, $j\in \{1,\dots ,N\}$, which represent the allocation bias effects regarding the *m* endpoints. Analogously to the univariate model of Hilgers et al. [19], we set up a multivariate linear model which incorporates the impact of allocation bias on the patient responses regarding the *m* endpoints by

1 $$\begin{aligned} \varvec{X_{j}}=\varvec{\mu _{E}}t_j+\varvec{\mu _{C}}(1-t_j)+\varvec{\tau _{j}}+\varvec{\epsilon _{j}}, \end{aligned}$$

where $\{\varvec{\epsilon _{j}}\}_{j\in \{1,\dots ,N\}}$ are independent and identically distributed error vectors with $\varvec{\epsilon _{j}}\sim \mathcal {N}({\textbf {0}},\Sigma )$, $j\in \{1,\dots ,N\}$. Note that $\textbf{0}\in \mathbb {R}^{m}$ is the vector with just zeros.

### Statistical analysis of multiple primary endpoints

Multiple primary endpoints are evaluated by analyzing the components separately with the following decision rule: Efficacy is established if at least one component shows a significant treatment effect. Using the statistical model (1), we obtain the *m* component-wise hypotheses

2 $$\begin{aligned} H_{0,k}: \mu _{C,k}=\mu _{E,k}\ vs.\ H_{1,k}: \mu _{C,k}\ne \mu _{E,k}, \end{aligned}$$

$k\in \{1,\dots ,m\}$, that can be combined to the global hypothesis

3 $$\begin{aligned} H_{0}: \varvec{\mu _{C}}=\varvec{\mu _{E}}\ vs.\ H_{1}: \varvec{\mu _{C}}\ne \varvec{\mu _{E}}. \end{aligned}$$

If all tests are conducted at the significance level $\alpha$, the probability of at least one erroneous rejection of the individual null hypotheses exceeds the nominal significance level $\alpha$. This probability is usually termed FWER and is commonly controlled by $\alpha$. Thus, to control the FWER the significance levels of the individual hypotheses need to be adjusted [6].

In 1967, Šidák introduced a less conservative procedure that uses the adjusted significance level $1-(1-\alpha )^{\frac{1}{m}}$ [8]. This adjustment guarantees the exact control of the FWER by the nominal significance level for independent endpoints in the unbiased case. However, the adjustments tend to be conservative for highly positively correlated endpoints [9].

### Statistical analysis of co-primary endpoints

Co-primary endpoints are evaluated simultaneously with a test strategy that follows an “all-or-none” decision rule [6]. The global test problem is given by

4 $$\begin{aligned} H_{0}: \bigcup \limits _{k=1}^{m} H_{0,k}\ vs.\ H_{1}: \bigcap \limits _{k=1}^{m} H_{1,k}. \end{aligned}$$

Therefore, this test is also often called intersection-union test. According to Berger [10] the *m* individual hypotheses $H_{0,k}\ vs.\ H_{1,k}$, $k\in \{1,\dots ,m\}$ are one-sided test problems. To mitigate the conservatism of the all-or-none test and to ensure the exact control of the significance level, we assume that the treatment effects of the endpoints are in the same direction: $\frac{\mu _{C,k}-\mu _{E,k}}{\sigma _k}\ge 0$, for all $k\in \{1,\dots ,m\}$. Then, the individuals hypotheses are defined by

5 $$\begin{aligned} H_{0,k}:\mu _{C,k}=\mu _{E,k}\ vs. \ H_{1,k}:\mu _{C,k} > \mu _{E,k}. \end{aligned}$$

To test the global test problem (4), the *m* individual hypotheses are tested at the nominal significance level $\alpha$. In contrast to multiple primary endpoints, no adjustment of the significance level is required [10].

### Randomization procedures

Randomization is used in clinical trials to minimize allocation bias and can be implemented by different procedures [19]. The most commonly used RPs in clinical trials are introduced below [20], for a more detailed description it is referred to Rosenberger and Lachin [21]:

- **Complete Randomization (CR):** Patients are assigned to the treatment and control group with a probability of a fair coin toss. Thus, the probability that a patient is allocated to the treatment group is always $\frac{1}{2}$. Note that the imbalance of group assignments is not controlled.
- **Efron Biased Coin (EBC(p)):** Patients are assigned with a probability equal to a biased coin toss that favors the less frequent allocations with probability $p \ge 0.5$.
- **Big Stick Design (BSD(b)):** The assignments are conducted with a probability equal to a fair coin toss until the maximum tolerated imbalance *b* between the treatment and control group is reached. Then the next patient is deterministically allocated to the group with fewer assignments.
- **Chen’s Urn Design (CHEN(p,b)):** Patients are assigned according to the EBC(p) until a maximum imbalance *b* between the treatment and control group is reached, then the next patient is deterministically allocated to the group with fewer assignments.
- **Maximal procedure (MP(b)):** Patients are allocated according to a randomization list which is uniformly selected from the subset of randomization lists generated by CR, which keeps the maximum tolerated imbalance *b*.
- **Random Allocation Rule (RAR):** The method randomly assigns half of patients to the treatment or control group.
- **Permuted Block Randomization (PBR(k)):** Patients are allocated in blocks of length *k*. Within these blocks, the allocation is according to RAR.

## Results

"Theoretical derivation" section provides the theoretical development for evaluating the influence of allocation bias in clinical trials with multiple endpoints. In "Simulation study" section, the impact of allocation bias is demonstrated through a simulation study, while in "Practical example" section, a clinical example is presented to further illustrate its effects.

### Theoretical derivation

In the following we derive theoretical results necessary for analyzing and evaluating the impact of allocation bias on the test decision. This encompasses the development of a biasing policy for clinical trials with multiple endpoints, as well as the derivation of formulas to compute the biased FWERs and T1Es of the misspecified analysis model. These measures quantify the bias effect on the test decision of the Šidák and all-or-none procedures, respectively, when the allocation bias is disregarded.

#### Biasing policy

To quantify the impact of allocation bias on the response of multiple endpoints in randomized, two-arm, parallel group clinical trials, we introduce a modified biasing policy based on Proschan [14]. We assume that the patient assignments are concealed but not masked. Thus, the researcher is assumed to be aware of previous allocations and can therefore forecast the next allocations. This is by definition, what we call third-order allocation bias. We assume that the investigator would prefer to enroll better responding patients in the treatment group and that a better responding patient is related to higher positive observed values of all $k\in \{1,\dots ,m\}$ continuous endpoints. If $\eta _k > 0$ describes the allocation bias effect for each endpoint $k\in \{1,\dots ,m\}$ then we can distinguish between good ($\eta _k$), neutral (0) and bad responders ($-\eta _k$). We assume that the researcher assigns the patients according to the convergence strategy introduced by Blackwell and Hodges in [13], where it is expected that the RP always tends to yield balanced allocations. Thus, if the previous allocations could be tracked to the control group, the next patient is more likely to be assigned to the treatment group and therefore a better-responding patient is enrolled. The next patient is more likely to be assigned to the control group if there are more assignments to the treatment group. In this case, a bad-responding patient is recruited. A neutral-responding patient is recruited if the assignments to both groups are balanced. Note that $N_E(j)$ and $N_C(j)$, $j\in \{1,\dots ,N\}$ are the number of allocations to the treatment and control group after *j* assignments, respectively. Then, the endpoint-specific allocation bias effect regarding the *k*-th endpoint in (1) are given by

6 $$\begin{aligned} \tau _{j,k}= \left\{ \begin{array}{ll} -\eta _k,& \text {if}\quad N_E(j-1) > N_C(j-1)\\ 0,& \text {if}\quad N_E(j-1)=N_C(j-1)\\ \eta _k,& \text {if}\quad N_E(j-1) < N_C(j-1) \end{array}\right. . \end{aligned}$$

#### Calculation of the Šidák adjusted FWER under bias

To derive a formula for calculating the biased FWER of the Šidák procedure, we consider the *m* test problems in (2) and assume that each of them is tested with a two-tailed t-test at a significance level of $\alpha ^*=1-(1-\alpha )^{\frac{1}{m}}$. Note that the mean response of the treatment and control group regarding the *k*-th endpoint is defined as $\overline{X}_{E,k}=\frac{1}{n_E}\sum \nolimits _{j=1}^{N}X_{j,k}t_j$ and $\overline{X}_{C,k}=\frac{1}{n_C}\sum \nolimits _{j=1}^{N}X_{j,k}(1-t_j)$, respectively. The test statistic, denoted by $S_k$, for the test problem $H_{0,k}\ vs.\ H_{1,k}$ is given by

7 $$\begin{aligned} S_k\!=\frac{\sqrt{\frac{n_En_C}{n_E+n_C}}(\overline{X}_{E,k}\!-\overline{X}_{C,k})}{\sqrt{\frac{1}{N}\!\left( \sum \limits _{j=1}^{N}t_j(X_{j,k}\!-\overline{X}_{E,k})^2+\sum \limits _{j=1}^{N}(1\!-t_j)(X_{j,k}\!-\overline{X}_{C,k})^2\right) }} \end{aligned}$$

Langer [15] demonstrates that for clinical trials with a single endpoint, the t-statistic applied to the model (1) follows a doubly non-central t-distribution under the null hypothesis. Thus, $S_k$, $k\in \{1,\dots ,m\}$ is under the null hypothesis $H_{0,k}$ doubly non-central t-distributed with non-centrality parameters

$$\begin{aligned} \delta _k & = \frac{1}{\sigma _k}\sqrt{\frac{n_En_C}{n_E+n_C}}\left( \mu _{E,k}-\mu _{C,k}+\overline{\tau }_{E,k}-\overline{\tau }_{C,k}\right) ,\\ \lambda _k & = \frac{1}{\sigma _k^2}\left( \sum \limits _{j=1}^{N}t_j(\tau _{j,k}-\overline{\tau }_{E,k})^2+\sum \limits _{j=1}^{N}(1-t_j)(\tau _{j,k}-\overline{\tau }_{C,k})^2\right) , \end{aligned}$$

where $\overline{\tau }_{E,k}=\frac{1}{n_E}\sum \limits _{j=1}^{N}\tau _{j,k}t_j$ and ${\overline{\tau }_{C,k}=\frac{1}{n_C}\sum \limits _{j=1}^{N}\tau _{j,k}(1-t_j)}$ for $k\in \{1,\dots ,m\}$. Thus, under the null hypothesis $H_{0,k}$ it holds

8 $$\begin{aligned} S_k\sim t^{\prime \prime }_{n_E+n_C-2,\delta _k,\lambda _k}, \end{aligned}$$

where $t^{\prime \prime }_{n_E+n_C-2,\delta _k,\lambda _k}$ denotes the doubly non-central t-distribution with $n_E+n_C-2$ degrees of freedom and non-centrality parameter $\delta _k$ and $\lambda _k$. The properties of the doubly non-central t-distribution are described in detail by Johnson, Kotz and Balakrishnan in [22].

Assuming that the *m* endpoints are uncorrelated and independent and using (8), the actual biased FWER for the allocation vector $\varvec{t}$ is calculated by

9 $$\begin{aligned} & P(\text {reject at least one } H_{0,k}|{\textbf {T}}=\varvec{t})\nonumber \\ & =1-P\left( \bigcap \nolimits _{k=1}^{m}\left( \vert S_k\vert \le t_{n_E+n_C-2}\left( 1-\frac{\alpha ^*}{2}\right) \right) |{\textbf {T}}=\varvec{t}\right) \nonumber \\ & {\mathop{=}^{\text{ind.}}_{\text{endp.}}} 1-\prod \nolimits _{k=1}^{m}\bigl [1-\bigl (F\left( t_{n_E+n_C-2}\left( \frac{\alpha ^*}{2}\right) ;n_E+n_C-2,\delta _k,\lambda _k\right) \nonumber \\ & +F\left( t_{n_E+n_C-2}\left( \frac{\alpha ^*}{2}\right) ;n_E+n_C-2,-\delta _k,\lambda _k\right) \bigr )\bigr ]. \end{aligned}$$

Here, $F(\cdot ; n_E+n_C-2,\delta _k,\lambda _k)$ denotes the distribution function of the doubly non-central t-distribution with $n_E+n_C-2$ degrees of freedom and non-centrality parameters $\delta _k$ and $\lambda _k$. Note that $t_{n_E+n_C-2}(\gamma )$ is the $\gamma$-quantile of the central t-distribution with ${n_E+n_C-2}$ degrees of freedom. To calculate the actual FWER without assuming independent endpoints, we need to transform the patient responses using a principle component analysis (PCA). The PCA transforms the patient responses using orthogonal transformations so that the components of the transformed patient responses, called principal components (PCs), are uncorrelated [23] and for normally distributed patient responses are also independent ([24], Chapter 3). Thereby, most of the variation in the patient responses should be retained. The orthogonal transformation of the patient responses is achieved through the orthonormal matrix $A\in \mathbb {R}^{m\times m}$, which consists of the orthonormal eigenvectors (EV) of $\Sigma$ [23]. The first column of *A* contains the EV to the largest eigenvalue of $\Sigma$, the second column contains the EV to the second largest eigenvalue of $\Sigma$, etc. Note that the transformed patient responses $\varvec{Y_{j}}:=A^T\varvec{X_{j}}$ , $j\in \{1,\dots ,N\}$ are also multivariate normally distributed according to

$$\begin{aligned} Y_{j}\sim \left\{ \begin{array}{ll} \mathcal {N}_m(A^T\varvec{\mu _{E}},\Lambda ),& \text {if}\quad t_j=1\\ \mathcal {N}_m(A^T\varvec{\mu _{C}},\Lambda ),& \text {if}\quad t_j=0 \end{array}\right. \end{aligned}$$

where $\Lambda \in \mathbb {R}^{m\times m}$ is the diagonal matrix of the eigenvalues, sorted in descending order. The transformed statistical model is according to (1) given by $\varvec{Y_{j}}=A^T\varvec{\mu _{E}}t_j+A^T\varvec{\mu _{C}}(1-t_j)+A^T\varvec{\tau _{j}}+A^T\varvec{\epsilon _{j}}$. Since the components of the PCA-transformed patient responses are independent, and the test problem (3) and $\widehat{H_{0}}: A^T\varvec{\mu _{C}}=A^T\varvec{\mu _{E}} \ vs.\ \widehat{H_{1}}: A^T\varvec{\mu _{C}}\ne A^T\varvec{\mu _{E}}$ are equivalent, the actual FWER for a given randomization list in clinical trials with positively correlated endpoints can be calculated similarly to uncorrelated endpoints, but using the transformed patient responses and the transformed statistical model.

#### Calculation of the type I error of the all-or-none procedure under bias

For the derivation of the formula to calculate the biased T1E of the all-or-none procedure, we assume that the global hypotheses (4) are tested at a nominal significance level $\alpha$ by testing the individual hypotheses (5) also at $\alpha$ with a one-tailed t-tests. The test statistics of these tests are given by $S_k$, $k\in \{1,\dots ,m\}$ in (7) and satisfy the property (8) under the one-sided null hypotheses. We assume ${R}=\bigcap \nolimits _{k=1}^{m}{R_k}$ is the event that $H_0$ is rejected in favor of $H_1$ and $R_k$ represents the rejection of the null hypothesis $H_{0,k}$. Then, the actual T1E for the allocation vector $\varvec{t}$ can be calculated according to Berger [10] by

$$\begin{aligned} P\left( \bigcap \nolimits _{k=1}^{m}{R_k}|{\textbf {T}}=\varvec{t}\right) = \underset{1\le k\le m}{max}P\left( S_k > t_{N_E+N_C-2}\left( 1-\alpha \right) |{\textbf {T}}=\varvec{t}\right) , \end{aligned}$$

where $t_{n_E+n_C-2}(1-\alpha )$ denotes the $(1-\alpha )$-quantile of the central t-distribution with $n_E+n_C-2$ degrees of freedom. Applying the property (8) leads us to following formula

10 $$\begin{aligned} & P\left( H_0 \text { is rejected}|{\textbf {T}}=\varvec{t}\right) \nonumber \\ & =\underset{1\le k\le m}{max}P\left( S_k > t_{n_E+n_C-2}\left( 1-\alpha \right) |{\textbf {T}}=\varvec{t}\right) \nonumber \\ & =\underset{1\le k\le m}{max}F\left( t_{n_E+n_C-2}\left( \alpha \right) ;n_E+n_C-2,-\delta _k,\lambda _k\right) . \end{aligned}$$

Note this computation is also applicable in the case of correlated endpoints.

### Simulation study

The impact of allocation bias on the test decisions is quantified by a simulation study. We simulate randomization lists using different RPs and calculate the actual biased FWER of the Šidák procedure and the actual biased T1E of the all-or-none approach using (9) and (10). To summarize the computed actual biased FWERs and T1Es across the randomization lists of the different RPs we use the mean as well as the probability of obtaining an FWER or T1E, respectively, below 5%, according to Hilgers et al. [19]. These probabilities are denoted by $P_{RP}(FWER\le 0.05)$ and $P_{RP}(T1E\le 0.05)$. If the FWER or T1E exceeds the 5% significance level, we refer to this as a not controlled error rate.

To compute the mean actual T1Es and actual FWERs as well as the probabilities $P_{RP}(FWER\le 0.05)$ and $P_{RP}(T1E\le 0.05)$ of the Šidák and all-or-none procedure we generate Monte Carlo samples of $r={100\,000}$ randomization lists for each RP considered. The examined RPs are CR, EBC(0.67), MP(3), BSD(3), RAR, PBR(4) and CHEN(2,0,67). We run the simulation with $m=2$ and $m=5$ multivariate standard normally distributed endpoints and focus on total sample sizes $N\in \{12,32,64\}$. We investigate homogeneous allocation bias effects, i.e., $\eta _k=\eta$ for all $k\in \{1,\dots ,m\}$, as proportion (1%, 5% and 10%) from the effect sizes. Table 1 shows these bias effects regarding the sample sizes *N*, the proportions $\nu$ and the effect sizes $E_N$. The different considered simulation settings for the Šidák and all-or-none procedure are summarized in Table 2.

**Table 1:** Examined allocation bias effects in the magnitude of $(\nu \cdot 100)\%$ of the effect size $E_N$, which depends on the sample size *N*

N	$\varvec{E}_{\varvec{N}}$	$\varvec{\nu }$	$\varvec{\eta }$
12	1.1795	0.01	0.01795
		0.05	0.08975
		0.1	0.1795
32	1.024	0.01	0.01024
		0.05	0.0512
		0.1	0.1024
64	0.711	0.01	0.00711
		0.05	0.03555
		0.1	0.0711

**Table 2:** Simulation settings to assess the impact of allocation bias on the test decisions of the Šidák and all-or-none procedures

Properties	Settings
Number of Endpoints	$m\in \{2,5\}$
Sample Size	$N\in \{12,32,64\}$
Distribution of Endpoints	multivariate standard normal
Allocation Bias	endpoint-specific allocation bias effects in the magnitude of 1%, 5% and 10% of the effect sizes.
RPs	CR, EBC(0.67), MP(3), BSD(3), RAR, PBR(4) and CHEN(2,0.67)

Computation of our simulation study are performed using the Software R-3.6.0 on the RWTH High Performance Computer Cluster using one core. The Monte Carlo samples of randomization lists of different RPs were generated using the R package randomizeR (v.1.4.2), published by Uschner et al. [18]. The computational time to simulate the actual FWERs of the Šidák procedure regarding $r= {100\,000}$ randomization lists for one clinical setting was approximately 48 seconds. The calculation of the actual T1E of the all-or-none procedure was completed in approximately 42 seconds. Hence, the calculations do not necessarily require the use of a high performance cluster, particularly if the quantification of allocation bias is performed only for a few clinical settings and bias effects.

#### Šidák procedure

**Fig. 1:**
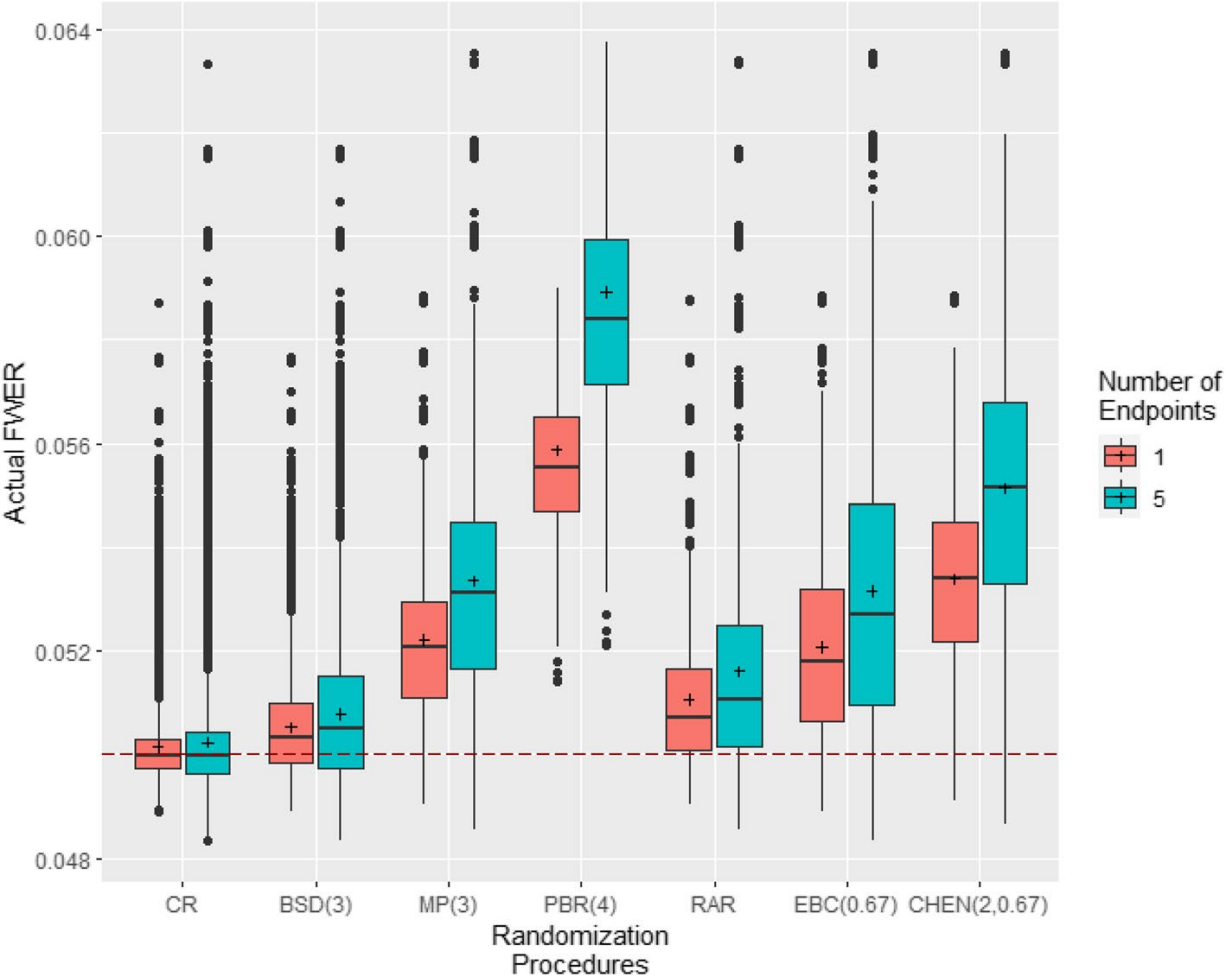
Comparison of allocation bias effects in clinical trials with single endpoints and multiple endpoints that are evaluatetd by the Šidák procedure. Simulations based on r= ${100\,000}$ randomization lists, $N=32$ patients and homogeneous allocation bias effects of $\eta =0.1 \cdot E_{32}$

We begin by evaluating the impact of allocation bias on the test decision of the Šidák procedure. Tables with the extended numerical results can be found in Section S1 of Additional file 1. Subsequently, we focus mainly on the analysis of homogeneous allocation bias effects and independent endpoints. Figure 1 illustrates the importance of studying the impact of allocation bias in multi-endpoint trials, as the error rates of multi-endpoint trials are more inflated under bias than those of single-endpoint trials.

In our first step, we examine the impact of allocation bias for varying numbers of endpoints. Table 3 shows the numerical results for the mean FWERs and the probability $P_{RP}(FWER\le 0.05)$ for clinical trials with $m=2$ and $m=5$ endpoints, a total sample size of $N=32$, and an allocation bias effect of 10% of the effect size regarding different RPs. These numerical results indicate that the number of endpoints has a minor effect on the impact of allocation bias on the summary measures, i.e., the mean FWER and $P_{RP}(FWER\le 0.05)$. Boxplots showing the distribution of the actual FWERs for the analyzed simulation settings are presented in Section S1 of Additional file 1, supporting these findings.

**Table 3:** Impact of allocation bias on the actual FWER for different numbers of endpoints (m) and RPs by using the Šidák procedure. Simulations based on r= ${100\,000}$ randomization lists, a sample size of $N=32$ and homogeneous allocation bias effect of $\eta =0.1\cdot E_{32}=0.1024$

m	RPs	FWER [mean]	$\varvec{P}_{\varvec{RP}}\varvec{(}\textbf{FWER}\varvec{\le 0.05)}$
2	CR	0.0502	0.55
	BSD(3)	0.0506	0.34
	MP(3)	0.0527	0.03
	PBR(4)	0.0572	0.00
	RAR	0.0513	0.18
	EBC(0.67)	0.0525	0.11
	CHEN(2,0.67)	0.0541	0.00
5	CR	0.0502	0.55
	BSD(3)	0.0508	0.34
	MP(3)	0.0534	0.03
	PBR(4)	0.0589	0.00
	RAR	0.0516	0.18
	EBC(0.67)	0.0532	0.11
	CHEN(2,0.67)	0.0551	0.00

Next, we focus on the impact of allocation bias for different sample sizes. The summary measures regarding various RPs for clinical trials with total sample sizes $N=12$, $N=32$ and $N=64$, $m=2$ endpoints and an allocation bias effect of 10% of the effect sizes are illustrated in Table 4. It can be noticed that with increasing sample size, the mean FWERs for CR, BSD(3), MP(3) and EBC(0.67) are stable, while the mean FWERs for CHEN(2,0.67) and PBR(4) increase and for RAR decrease. The probability of maintaining the 5% level decreases with increasing sample size for all RPs. The best control of the nominal significance level is provided by CR and BSD(3). Boxplots showing the distribution of actual FWERs for different sample sizes are presented in Section S1 of Additional file 1, illustrating that the variability of actual FWERs decreases with increasing sample size.

**Table 4:** Impact of allocation bias on the actual FWER for different sample sizes (N) and RPs by using the Šidák procedure. Simulations based on r=${100\,000}$ randomization lists, $m=2$ endpoints and homogeneous allocation bias effects of $\eta =0.1\cdot E_N$

N	RPs	FWER [mean]	$\varvec{P}_{\varvec{RP}}\varvec{(}\textbf{FWER}\varvec{\le 0.05)}$
12	CR	0.0503	0.61
	BSD(3)	0.0506	0.52
	MP(3)	0.0531	0.18
	PBR(4)	0.0562	0.03
	RAR	0.0527	0.24
	EBC(0.67)	0.0524	0.31
	CHEN(2,0.67)	0.0532	0.19
32	CR	0.0502	0.55
	BSD(3)	0.0506	0.34
	MP(3)	0.0527	0.03
	PBR(4)	0.0572	0
	RAR	0.0513	0.18
	EBC(0.67)	0.0525	0.11
	CHEN(2,0.67)	0.0541	0
64	CR	0.0501	0.54
	BSD(3)	0.0508	0.15
	MP(3)	0.0527	0
	PBR(4)	0.0575	0
	RAR	0.0507	0.18
	EBC(0.67)	0.0526	0.03
	CHEN(2,0.67)	0.0545	0

**Fig. 2:**
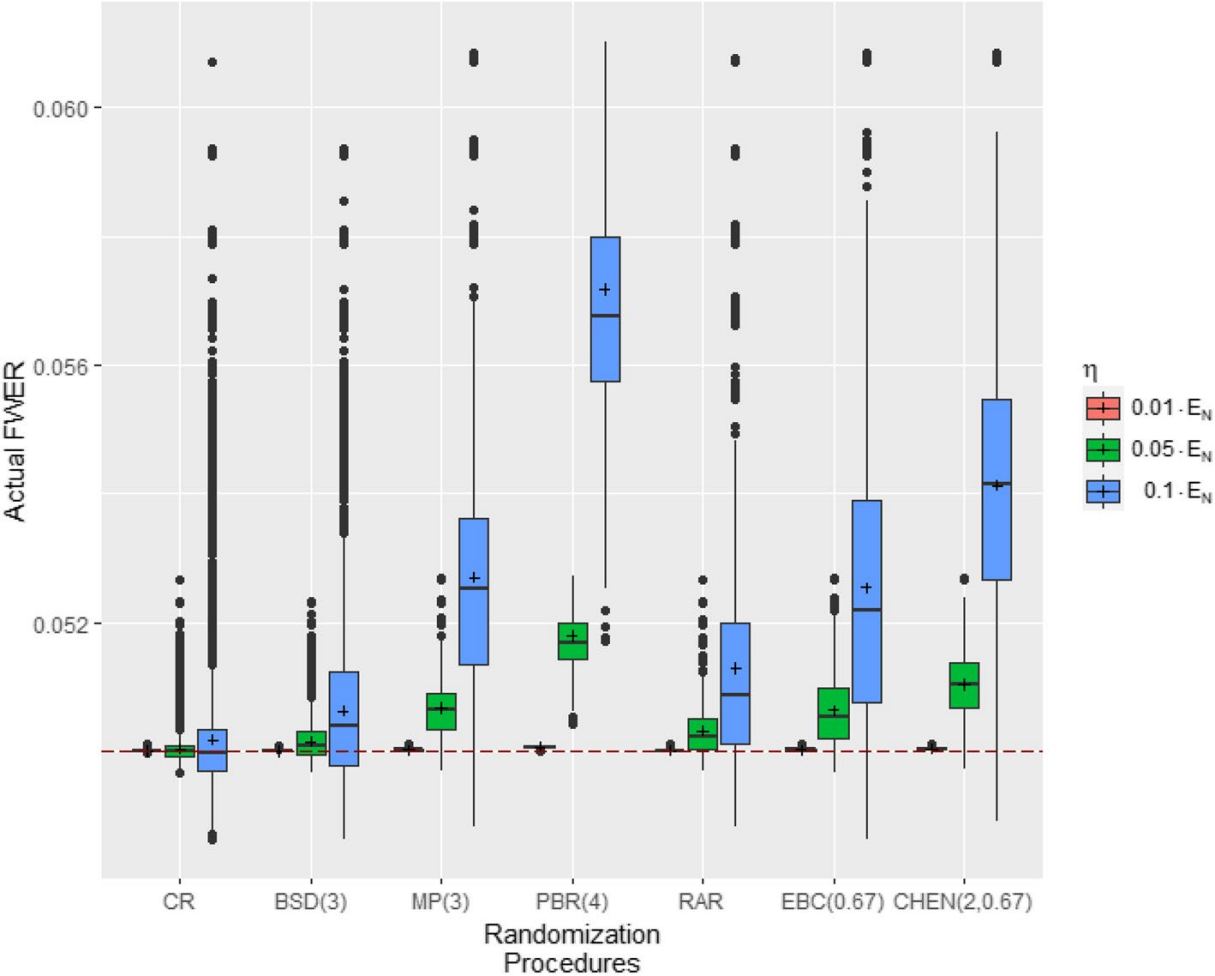
Distribution of the actual FWER of the Šidák procedure for different homogeneous allocation bias effects. Simulations based on r= ${100\,000}$ randomization lists, $m=2$ endpoints, $N=32$ patients and homogeneous allocation bias effects of $\eta =\nu \cdot E_{32}$ with $\nu \in \{0.01,0.05,0.1\}$

To investigate the effect of different allocation bias effects Fig. 2 shows the distribution of the actual FWER and Table 5 outlines the mean FWERs and the probabilities $P_{RP}(\text {FWER}\le 0.05)$ of clinical trials with $m=2$ endpoints, $N=32$ patients, for bias effects of 1%, 5%, and 10% of the effect size regarding different RPs. We observe that increasing allocation bias effects result in increased variability of the actual FWERs and inflated mean FWERs. The shift is most pronounced for CHEN(2,0.67) and PBR(4), while it is less pronounced for CR and BSD(3). Table 5 indicates that, instead of increasing allocation bias effects, the probabilities $P_{RP}(\text {FWER}\le 0.05)$ remain at the same level. Only for CR the probability of maintaining the significance level is above 50%. This is followed by BSD(3), whose probability is 34%. For CHEN(2,0.67) and PBR(4), the control of the significance level is in the presence of the examined bias effects not ensured.

**Table 5:** Impact of different homogeneous allocation bias effects on the actual FWER of the Šidák procedure regarding different RPs. Simulations based on r= ${100\,000}$ randomization lists, $m=2$ endpoints, $N=32$ patients and homogeneous allocation bias effects of $\eta =\nu \cdot E_{32}$ with $\nu \in \{0.01,0.05,0.1\}$

Allocation bias effect $\varvec{[\eta ]}$	RPs	FWER [mean]	$\varvec{P}_{\varvec{RP}}\varvec{(}\textbf{FWER}\varvec{\le 0.05)}$
$0.01\cdot E_{32}$	CR	0.0500	0.55
	BSD(3)	0.0500	0.34
	MP(3)	0.0500	0.03
	PBR(4)	0.0501	0.00
	RAR	0.0500	0.18
	EBC(0.67)	0.0500	0.11
	CHEN(2,0.67)	0.0500	0.00
$0.05\cdot E_{32}$	CR	0.0500	0.55
	BSD(3)	0.0502	0.34
	MP(3)	0.0507	0.03
	PBR(4)	0.0518	0.00
	RAR	0.0503	0.18
	EBC(0.67)	0.0506	0.11
	CHEN(2,0.67)	0.0510	0.00
$0.1\cdot E_{32}$	CR	0.0502	0.55
	BSD(3)	0.0506	0.34
	MP(3)	0.0527	0.03
	PBR(4)	0.0572	0.00
	RAR	0.0513	0.18
	EBC(0.67)	0.0525	0.11
	CHEN(2,0.67)	0.0541	0.00

**Fig. 3:**
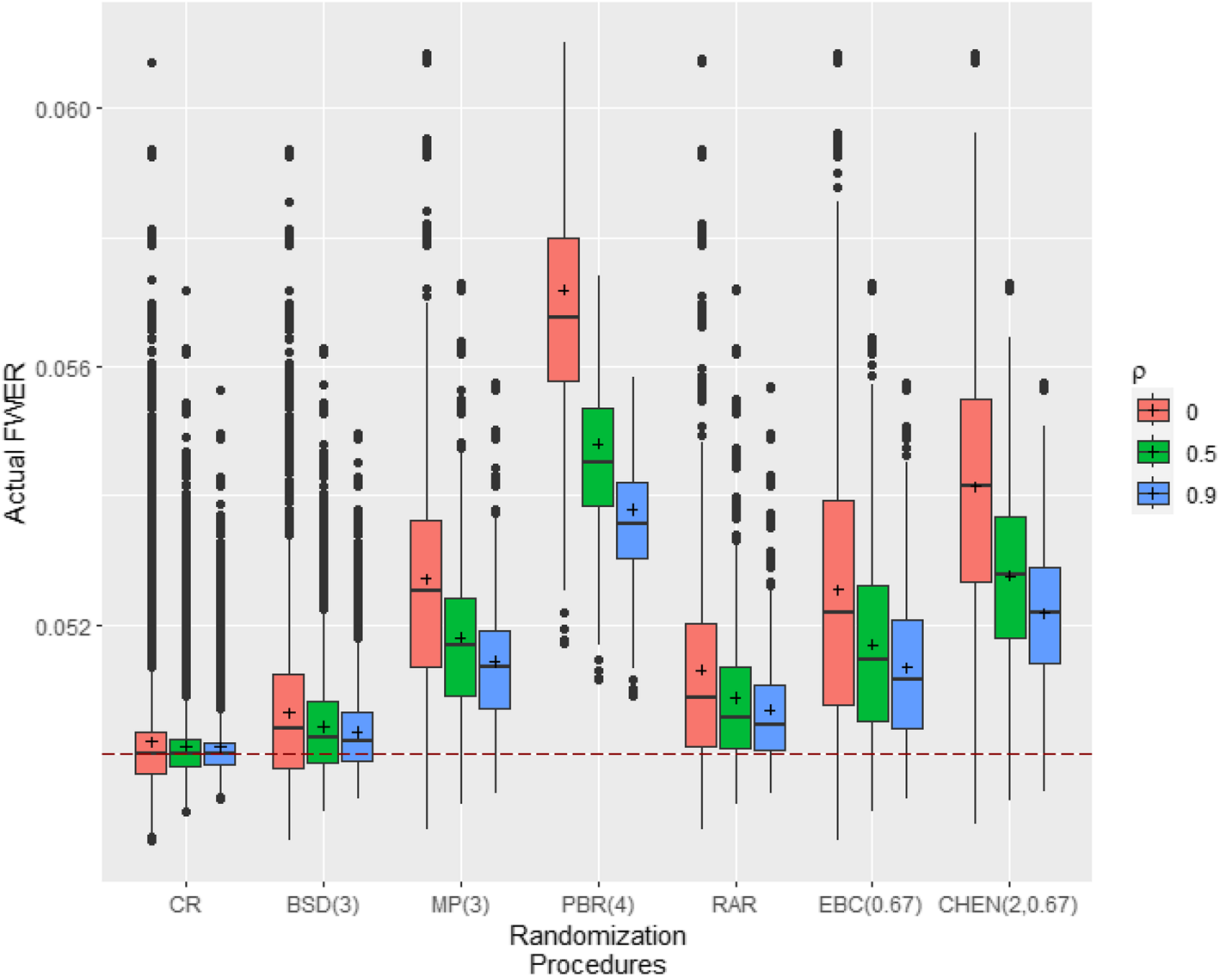
Distribution of the actual FWER of the Šidák procedure for homogeneous allocation bias effects in clinical trials with correlated endpoints. Simulations based on r= ${100\,000}$ randomization lists, $m=2$ correlated endpoints according to $R_{\rho }$, sample size $N=32$ and homogeneous allocation bias effects of $\eta =0.1\cdot E_{32}$

Using the approach in "Calculation of the Šidák adjusted FWER under bias" section we analyze the impact of allocation bias for endpoints that are correlated according to the correlation matrix

$$\begin{aligned} R_{\rho }= \left( \begin{array}{ccccc} 1& \rho & \cdots & \cdots & \rho \\ \rho & 1& \rho & \cdots & \rho \\ \rho & & \ddots & & \vdots \\ \vdots & & & \ddots & \rho \\ \rho & \cdots & \cdots & \rho & 1 \end{array}\right) \in \mathbb {R}^{m\times m} \end{aligned}$$

with $\rho \in [-1,1]$. Figure 3 shows the distribution of the actual FWER for different RPs in a clinical trial with $m=2$ endpoints that are positively correlated according to * Rρ* with $\rho \in \{0,0.5,0.9\}$, $N=32$ patients and homogeneous allocation bias effects of 10% of the effect size. Increasing positive correlation between endpoints leads to decreasing variability of the actual FWERs and decreasing mean FWERs. Thus, uncorrelated endpoints are the worst-case scenario for homogeneous allocation bias effects and positively correlated endpoints. Note that this does not necessarily extends to heterogeneous allocation bias effects. The impact of allocation bias on the actual FWER for correlated endpoints is, as for uncorrelated endpoints, strongly dependent on the allocation bias effects chosen for the individual endpoints. A detailed overview of the simulation results for positively correlated endpoints can be found in Section S1 of Additional file 1. Previously, we focused on homogeneous bias effects. When considering heterogeneous bias effects, we observe that larger sums of endpoint-specific bias effects lead to stronger inflated error rates. Section S1 of Additional file 1 provides additional simulation results for heterogeneous bias effects.

#### All-or-none procedure

In the following, we focus on the impact of allocation bias in clinical trials with co-primary endpoints that are analyzed by the all-or-none procedure, and show the results of the mean actual T1Es and the probabilities $P_{RP}(\text {T1E}\le 0.05)$ for several simulation scenarios. Due to same directed treatment effects, the significance level is exactly controlled in the unbiased case. Tables with extended results and boxplots of the numeric results presented in Tables 6 and 7 are given in Section S2 of Additional file 1.

We start by analyzing the effect of different numbers of components of the co-primary endpoint on the impact of allocation bias. Table 6 reports the results for clinical trials with $m=2$ and $m=5$ components, $N=32$ patients, and homogeneous allocation bias effects of 10% of the effect sizes for several RPs. We observe that the numerical results in clinical trials with co-primary endpoints with $m=2$ and $m=5$ components are identical. This is mainly due to the fact that the maximum of the doubly non-central t-distribution over all components in the formula (10) remains the same when using equally distributed components. Thus, if the components are equally distributed, the number of multiple endpoints does not affect the actual T1E.

**Table 6:** Impact of allocation bias on the actual T1E for different numbers of endpoints (m) and RPs by using the all-or-none procedure. Simulations based on r= ${100\,000}$ randomization lists, a sample size of $N=32$ patients and homogeneous allocation bias effects $\eta =0.1\cdot E_N=0.1024$ for all endpoints

m	RPs	T1E [mean]	$\varvec{P}_{\varvec{RP}}(\textbf{T1E}\varvec{\le 0.05)}$
2	CR	0.0555	0.09
	BSD(3)	0.0598	0.02
	MP(3)	0.0676	0.00
	PBR(4)	0.0790	0.00
	RAR	0.0620	0.00
	EBC(0.67)	0.0664	0.01
	CHEN(2,0.67)	0.0717	0.00
5	CR	0.0555	0.09
	BSD(3)	0.0598	0.02
	MP(3)	0.0676	0.00
	PBR(4)	0.0790	0.00
	RAR	0.0620	0.00
	EBC(0.67)	0.0664	0.01
	CHEN(2,0.67)	0.0717	0.00

We continue with the analysis of the effect of different sample sizes. Table 7 provides the numerical results of the summary measures regarding different RPs for clinical trials with total sample sizes $N=12$, $N=32$ and $N=64$, co-primary endpoints with $m=2$ components and homogeneous allocation bias effects of 10% of the effect sizes. The mean T1Es decrease regarding all randomization procedures except CHEN(2,0.67) for increasing sample sizes. Note that the probabilities $P_{RP}(\text {T1E}\le 0.05)$ regarding the different RPs remain mostly unaffected by varying sample sizes. Consequently, the sample size has no considerable effect on the probability of controlling the actual T1E in the presence of allocation bias. Notice that regardless of sample size and RP, the likelihood that T1E failing the nominal significance level is more than 90%. Overall, the control of the T1Es by the nominal significance level in the presence of allocation bias is weak.

**Table 7:** Impact of allocation bias on the actual T1E for different sample sizes and RPs by using the all-or-none procedure. Simulations based on r= ${100\,000}$ randomization lists, $m=2$ endpoints and homogeneous allocation bias effects of $\eta =0.1\cdot E_N$

N	RPs	T1E [mean]	$\varvec{P}_{\varvec{RP}}\varvec{(}\textbf{T1E}\varvec{\le 0.05)}$
12	CR	0.0589	0.09
	BSD(3)	0.0611	0.06
	MP(3)	0.0705	0.00
	PBR(4)	0.0792	0.00
	RAR	0.0688	0.00
	EBC(0.67)	0.0677	0.03
	CHEN(2,0.67)	0.0712	0.01
32	CR	0.0555	0.09
	BSD(3)	0.0598	0.02
	MP(3)	0.0676	0.00
	PBR(4)	0.0790	0.00
	RAR	0.0587	0.00
	EBC(0.67)	0.0664	0.01
	CHEN(2,0.67)	0.0717	0.00
64	CR	0.0539	0.09
	BSD(3)	0.0599	0.00
	MP(3)	0.0668	0.00
	PBR(4)	0.0790	0.00
	RAR	0.0587	0.00
	EBC(0.67)	0.0662	0.00
	CHEN(2,0.67)	0.0720	0.00

**Fig. 4:**
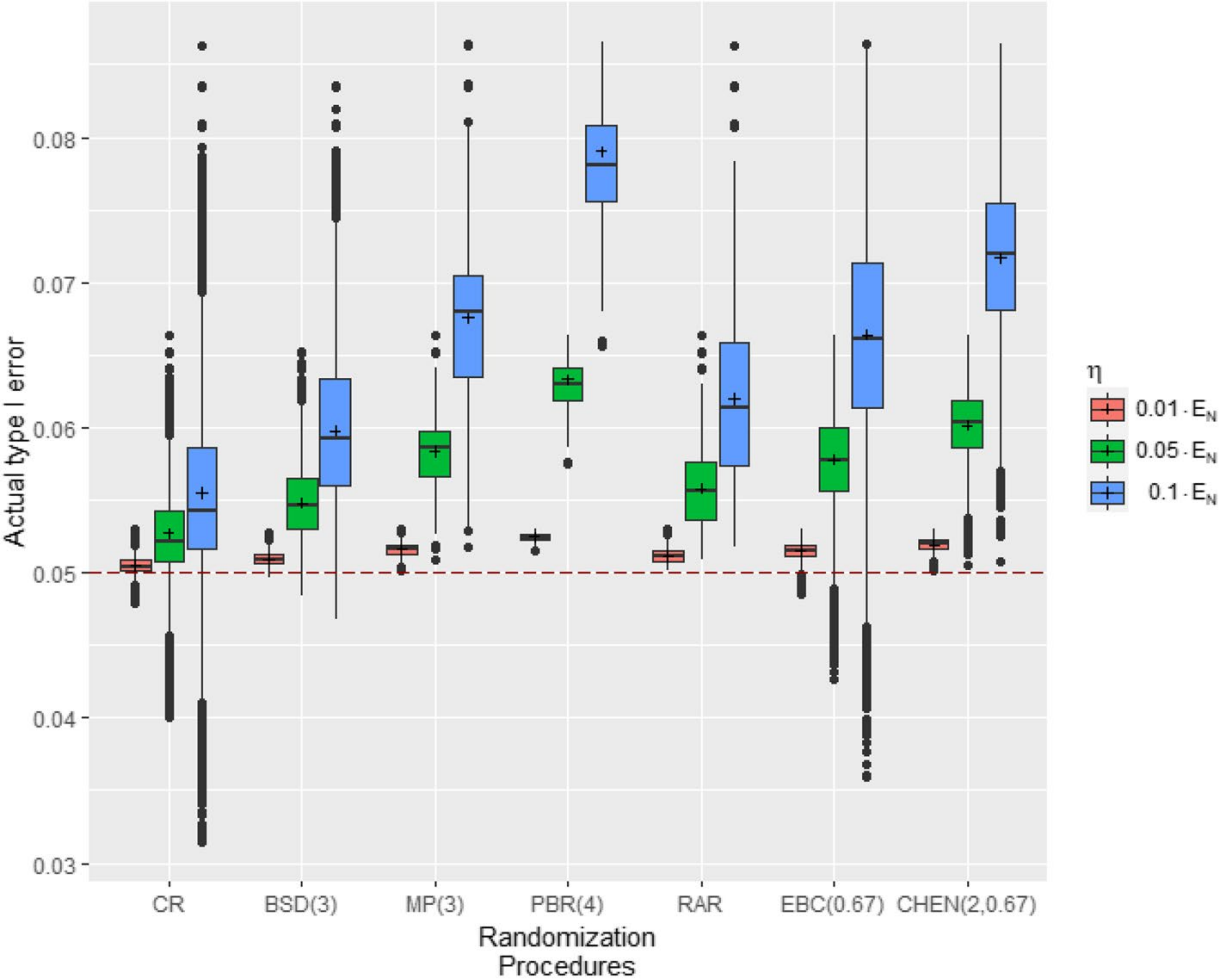
Distribution of the actual T1E of the all-or-none procedure for different homogeneous allocation bias effects. Simulations based on r= ${100\,000}$ randomization lists, $m=2$ endpoints, $N=32$ patients and homogeneous allocation bias effects of $\eta =\nu \cdot E_{32}$ with $\nu \in \{0.01,0.05,0.1\}$

This is also supported by considering Fig. 4 and the values in Table 8. Figure 4 shows the distribution of T1E regarding different RPs for homogeneous allocation bias effects of 1%, 5% and 10% of the effect size in a clinical trial with co-primary endpoints with $m=2$ components and $N=32$ patients. Table 8 shows for the identical settings the numerical values of the mean T1Es and probabilities $P_{RP}(\text {T1E}\le 0.05)$. We observe that with increasing allocation bias effects, the variability and the mean values of the T1Es increase independently of the RP. The probabilities $P_{RP}(\text {T1E}\le 0.05)$ in Table 8 indicate that even small allocation bias effects cause that the actual T1E to be insufficiently controlled by the nominal significance level. For CR, controlling the T1E by the nominal significance level is best with a probability of 8%.

Previously, we focused on homogeneous bias effects. Analyzing heterogeneous bias effects show that the inflation of the error rates depends on the sum of the endpoint-specific bias effects. Thus, increasing sums of endpoint-specific bias effects are associated with stronger inflation of the error rates. Simulation results for heterogeneous bias effects are presented in Section S2 in Additional file 1.

**Table 8:** Impact of different homogeneous allocation bias effects on the actual T1E of the all-or-none procedure regarding different RPs. Simulations based on r= ${100\,000}$ randomization lists, $m=2$ endpoints, $N=32$ patients and homogeneous allocation bias effects $\eta =\nu \cdot E_{32}$ with $\nu \in \{0.01,0.05,0.1\}$

Allocation bias effect [$\varvec{\eta }$]	RPs	T1E [mean]	$\varvec{P}_{\varvec{RP}}\varvec{(}\textbf{T1E}\varvec{\le 0.05)}$
$0.01\cdot E_{32}$	CR	0.0505	0.08
	BSD(3)	0.0509	0.01
	MP(3)	0.0516	0.00
	PBR(4)	0.0525	0.00
	RAR	0.0511	0.00
	EBC(0.67)	0.0515	0.01
	CHEN(2,0.67)	0.0519	0.00
$0.05\cdot E_{32}$	CR	0.0527	0.08
	BSD(3)	0.0548	0.01
	MP(3)	0.0584	0.00
	PBR(4)	0.0633	0.00
	RAR	0.0558	0.00
	EBC(0.67)	0.0578	0.01
	CHEN(2,0.67)	0.0602	0.00
$0.1\cdot E_{32}$	CR	0.0555	0.09
	BSD(3)	0.0598	0.02
	MP(3)	0.0676	0.00
	PBR(4)	0.0790	0.00
	RAR	0.0620	0.00
	EBC(0.67)	0.0664	0.01
	CHEN(2,0.67)	0.0717	0.00

### Practical example

In the following, we will illustrate how the approach can be used to formulate scientific arguments for the selection of a RP according to the ERDO template [19]. Therefore, we will use information from the multi-center randomized EPISTOP trial to plan a new clinical trial [25]. The EPISTOP trial compares a conventional vigabatrin therapy, initiated after the first electrographic or clinical seizure, with a preventive approach that commences prior to the first seizure in infants diagnosed with Tuberous sclerosis complex (TSC) [26] without a seizure history. TSC is a rare genetic disorder that not only causes forms of epilepsy but also affects the neuronal and intellectual development of infants. We will design a future hypothetical clinical trial to investigate the impact of preventive versus conventional treatment with vigabatrin on the neuronal development of infants aged $\le 4$ months diagnosed with TSC, but without a history of seizures. Subsequently, we will discuss the study layout, statistical model, and potential bias in the new trial. The trial will be a randomized two-arm, single-center study with multiple continuous endpoints. It will follow a 1:1 allocation ratio and exclude interim and adaptive analysis. To assess the effects of conventional versus preventive treatment, we will employ the Bayley Scales of Infant Toddler Development (BSID) [27] to evaluate the neural and intellectual development of the infants. These scales describe the early development of infants and can be divided into a cognitive ($BSID_{cognitive}$), a language ($BSID_{language}$), a motor ($BSID_{motor}$), an adaptive behavior ($BSID_{ab}$) and a social-emotional ($BSID_{se}$) scale. Each of these scales form a component of the multiple primary endpoint and are individually compared between preventive and conventional treatment at 24 months. The null hypotheses of no differences between the preventive and conventional treatment regarding these components are tested individually by t-tests for homogeneous variances on Šidák adjusted significance levels. Note that the caregiver and investigators are blinded to the EEG data and allocation of the infants to the treatment groups. The infants’ allocation are also hidden from the neuropsychologists who perform neurological tests and the assessors of the EEGs. Based on the results of the EPISTOP trial regarding the 27 randomized infants the mean scales and standard deviations of the conventional treatment and preventive treatment group and the resulting effect sizes concerning the *BSID* scores are given in Table 9. The correlation matrix of both groups is according to the EPISTOP data estimated by

$$\begin{aligned} R= \left( \begin{array}{ccccc} 1& 0.79& 0.86& 0.22& 0.48\\ 0.79& 1& 0.77& 0.30& 0.57\\ 0.86& 0.77 & 1& 0.15& 0.46\\ 0.22& 0.30& 0.15 & 1& 0.46\\ 0.48& 0.57& 0.46& 0.46 & 1 \end{array}\right) \end{aligned}$$

**Table 9:** Standard deviations, mean values and effect sizes of the *BSID* scores of the conventional treatment group (CT) and the preventive treatment group (PT) based on the results of the 27 randomized patients in the EPISTOP trial

Scales	Group	Standard deviation	Mean	Effect size
$BSID_{cognitive}$	CT	15.78	74.09	0.114
	PT	15.63	72.31	
$BSID_{language}$	CT	16.36	71.45	0.509
	PT	12.05	64.23	
$BSID_{motor}$	CT	16.35	74.27	0.315
	PT	13.79	69.54	
$BSID_{ab}$	CT	23.63	75.09	0.361
	PT	19.25	67.38	
$BSID_{se}$	CT	29.52	101.82	1.034
	PT	14.67	78.33	

If we assume a power of 80% and a significance level of $\alpha =0.05$, the required sample size is estimated in addition to a dropout of 10% by $N=24$ [28]. For the allocation bias we assume endpoint-specific allocation bias effects in the magnitude of 1% and 10% of the effect sizes. The ERDO template aims to find the optimal RP for a clinical trial. Therefore, we consider CR, RAR, BSD, EBC, MP, CHEN, and PBR and evaluate them by the metrics defined in the simulation section, i.e., the mean FWERs and the probabilities $P_{RP}(\text {FWER}\le 0.05)$ determined by Monte Carlo samples of ${100\,000}$ randomization lists. Table 10 shows the numerical results of these metrics. For the mean FWERs, we observe comparable results for all RPs. However, if we consider the probabilities $P_{RP}(\text {FWER}\le 0.05)$, we see that only for CR the probabilities that the FWER is controlled by the nominal significance level despite allocation bias are above 50%. Whereas, for PBR(4), the FWERs are slightly controlled. The most promising RPs that are identified on the basis of the probabilities $P_{RP}(\text {FWER}\le 0.05)$ for mitigating bias are CR and BSD(3).

**Table 10:** Impact of different allocation bias effects $\eta =(\eta _1,\dots ,\eta _5)^T$ on the actual FWERs for different RPs by using the Šidák procedure. Simulations based on r= ${100\,000}$ randomization lists, $N=24$ patients and $\sigma =(15.70, 14.19, 15.02, 21.37, 22.71)^T$

Allocation bias effect	RPs	FWER [mean]	$\varvec{P}_{\varvec{RP}}(\textbf{FWER}\varvec{\le 0.05)}$
$$0.01\cdot \left( \begin{array}{c} 0.114\\ 0.509\\ 0.315\\ 1.034\\ 0.361\end{array}\right)$$	CR BSD(3) MP(3) PBR(4) RAR EBC(0.67) CHEN(2,0.67)	0.0500 0.0500 0.0500 0.0500 0.0500 0.0500 0.0500	0.55 0.37 0.06 0 0.15 0.16 0.02
$$0.1\cdot \left( \begin{array}{c} 0.114\\ 0.509\\ 0.315\\ 1.034\\ 0.361\end{array}\right)$$	CR BSD(3) MP(3) PBR(4) RAR EBC(0.67) CHEN(2,0.67)	0.0500 0.0500 0.0500 0.0500 0.0500 0.0500 0.0500	0.56 0.37 0.06 0 0.19 0.16 0.02

It should be noted, that the assumed effects, e.g. the amount of allocation bias, used in the planning phase of a clinical trial might deviate from the actually observed one after trials conduct. We conclude that allocation bias affects the error rates and can be best mitigated by relaxing the balance between the preventive and conservative group sizes. Overall, this example suggests that allocation bias should be considered in the design phase of clinical trials to increase validity.

## Discussion

Randomization is one of the most important design features of a clinical trial to prevent allocation bias [19]. Selection of a randomization procedure (RP) on scientific arguments, i.e. quantification of the potential to mitigate bias, increases the validity of a clinical trial. In clinical practice, the choice of a RP is usually in favour of permuted block randomization (PBR) due to its simple implementation and familiarity [20, 29]. However, this selection is usually not based on scientific recommendations and arguments. This paper aims to raise awareness of the selection of a RP on the validity of a clinical trial by mitigating bias.

We have extended the methods of the ERDO template [19] to provide a more scientific justification for the selection of a RP in clinical trials with multiple endpoint. Therefore, we introduced an allocation biasing policy for clinical trials with multiple primary and co-primary endpoints, and derived formulas to calculate the error rates depending on different RP when the endpoints are evaluated using the Šidák or the all-or-none procedure. We applied these derivations in a simulation study to quantify the impact of allocation bias on the test decision of the Šidák and the all-or-none procedure. We found that allocation bias leads to inflation of the actual FWER and T1E. The amount of inflation depends on the magnitude of homogeneous allocation bias effects and the chosen RP. Interestingly, the number of components of the multiple primary and co-primary endpoints, as well as small sample sizes, has no considerable effect on the impact of homogeneous allocation bias.

The most promising RPs that have been identified for mitigating bias by using the Šidák procedure are CR and BSD because the FWERs regarding these procedures are less sensitive to allocation bias and better controlled by the nominal significance level.

In clinical trials with co-primary endpoints that are analyzed using the all-or-none procedure, the control of the actual T1E by the nominal significance level for all endpoints is deficient. The best way to prevent allocation bias is CR.

The Šidák and all-or-none procedure are completely different, as the research questions mirror different clinical situations. We found that the Šidák procedure is less sensitive to allocation bias than the all-or-none procedure considering the biased FWER and T1E, respectively. Note that the simulations were restricted to clinical trials with multiple primary and co-primary endpoints that are evaluated by the Šidák and all-or-none procedure. To fully investigate the effect of allocation bias, it is necessary to study more complicated testing procedures such as the closed test procedure or other significance level adjustments such as the Bonferroni adjustment [6] or the adjustment according to Sankoh [30]. This can be achieved by adapting the introduced model to the specific test procedure. We focused on the Šidák procedure rather than the Bonferroni procedure since the Šidák procedure is less conservative. Note that Sankohs’ approach is not commonly used, but our method can be extended to this approach. Also the consideration of other types of multiple endpoints as composite or multi-component endpoints should be subject of further research [5]. The impact of allocation bias was quantified only for a limited number of RPs and parameters. To gain more comprehensive insights, additional parameters of the RPs may be taken into account. The current selection is motivated by the different properties of the RPs. Of course, the approach presented so far for choosing a suitable RP is based only on the impact of allocation bias on the test decision, but overall the selection process should also consider other aspects of the trial design, such as power, other types of bias, etc. It should be noted that mainly small and homogeneous endpoint-specific allocation bias effects were considered in the simulations. A detailed analysis of heterogeneous endpoint-specific allocation bias effects are subject to further research. In practice, it is challenging to determine the endpoint-specific allocation bias effects $\eta _k$, $k\in \{1,\dots , m\}$, since they are often unknown in the planning phase of a clinical trial. To overcome this, the bias effects could be approximated based on clinical experience, or linked to the effect sizes determined from previously published data, e.g. from trials in similar diseases. Overall, we can conclude that the presented methodology can be used for selecting the RP of a clinical trial with multiple primary endpoints or co-primary endpoints based on the criteria of mitigating allocation bias. In the future, we aim to extend this methodology to more general models that use approaches to detect a global treatment effect in a clinical trial with multiple endpoints by aggregating the multivariate endpoint data into a single score or rank at the subject level, a single composite endpoint, or a univariate test statistic. In addition, multiple endpoints with both continuous or binary components should be examined next. Besides considering allocation bias in clinical trials with multiple endpoints, bias effects should also investigated for other trial designs, such as adaptive trials or trials with unbalanced allocation.

### Conclusion

Ignoring allocation bias affects the test decisions of a clinical trial and the selection of an appropriate RP is one of the most important tool to minimize the effect of allocation bias. We found that RPs with relaxation of the final balance, i.e., the final balance of allocations to the control and treatment arm, are useful for mitigating allocation bias in clinical trials with multiple primary and co-primary endpoints that are evaluated using the Šidák and all-or-none procedures, respectively. Thus, RPs as CR and BSD are more preferable to protect against allocation bias.

## Supplementary Information

Additional file 1.

## Data Availability

Data from the EPISTOP trial will not be shared because patients’ informed consent at the time of recruitment to the trial does not include open data access. The code to reproduce the results of the simulation study are available in the “The impact of allocation bias on test decisions in clinical trials with multiple endpoints using multiple testing strategies” repository, https://github.com/IMSA-RWTH/allocation-bias-multiple-endpoints.
