# Supplementary material for: The impact of allocation bias on test decisions in clinical trials with multiple endpoints using multiple testing strategies
Source: BMC Med Res Methodol. 2024 Sep 30;24:223. doi: 10.1186/s12874-024-02335-x (PMC11441119; doi:10.1186/s12874-024-02335-x)
Supplement: Supplementary file 1 — Additional file 1. [file 12874_2024_2335_MOESM1_ESM.pdf]

The impact of allocation bias on test decisions in clinical trials with multiple endpoints using multiple testing strategies

Supplementary Material

Contents

S1: Detailed simulation results regarding the impact of allocation bias by using the Šidák procedure **1**

S2: Detailed simulation results regarding the impact of allocation bias by using the all-or-none procedure **11**

S3: Impact of allocation bias for the Šidák versus Bonferroni procedure **17**

S1: Detailed simulation results regarding the impact of allocation bias by using the Šidák procedure

In the following, we show detailed results of the simulation study introduced in Section 3.2. The simulation study analyzes the impact of allocation bias on the actual FWER of the Šidák procedure regarding different randomization procedures (RPs).

Boxplots of the simulation results regarding the Šidák procedure presented in the manuscript

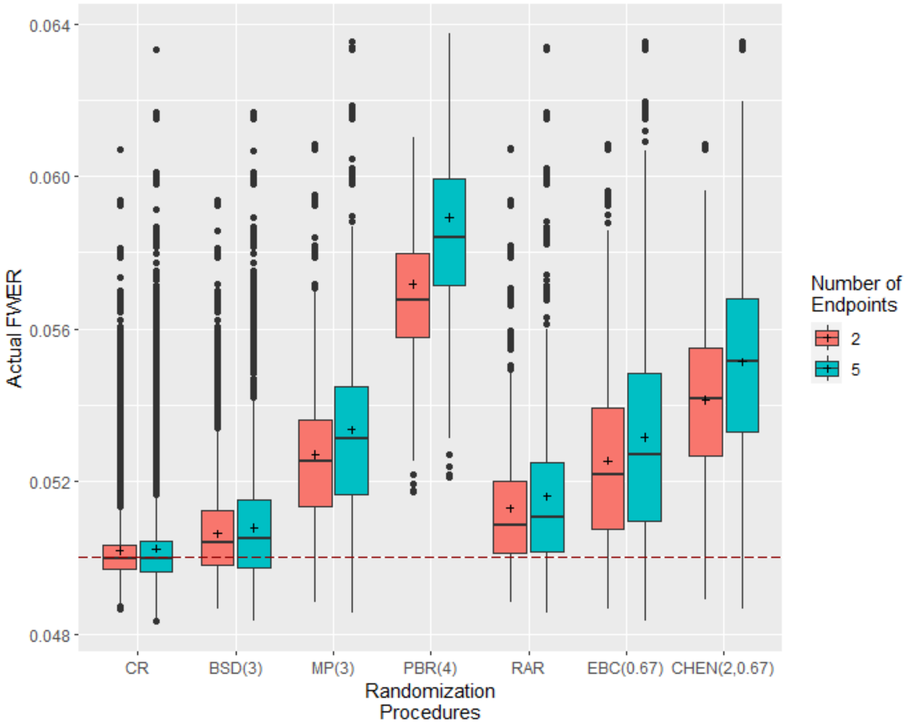

Figure S1: Distribution of the actual FWERs of the Šidák procedure for different numbers of endpoints. Simulations based on r=100 000 randomization lists, a sample size of $N = 32$ patients and homogeneous allocation bias effects $\eta = 0.1 \cdot E_N = 0.1024$ for all endpoints.

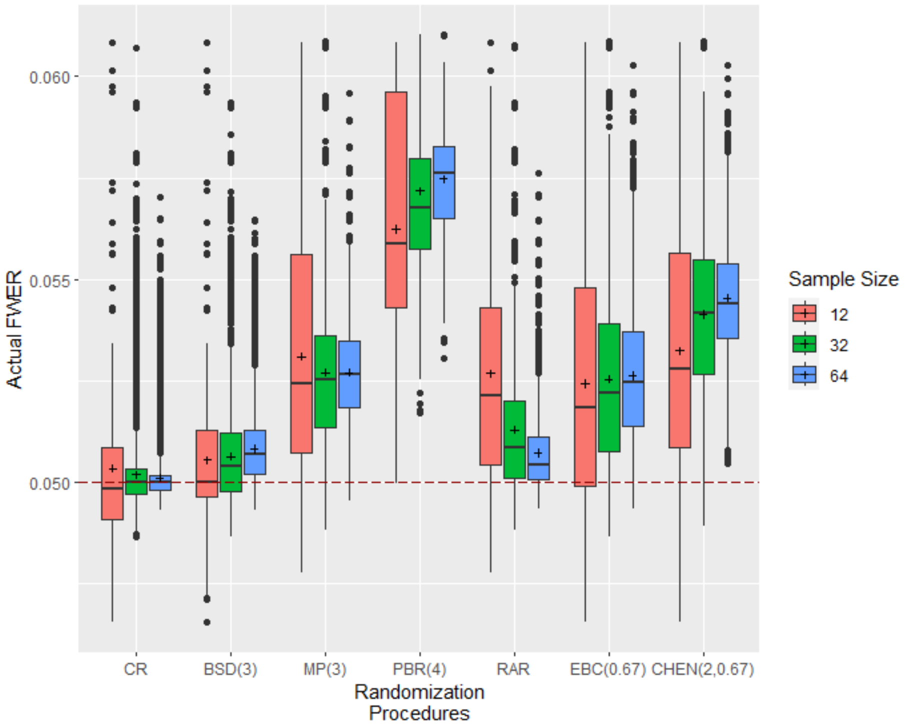

Figure S2: Distribution of the actual FWERs of the Šidák procedure for different sample sizes. Simulations based on r=100 000 randomization lists, $m = 2$ endpoints and homogeneous allocation bias effects of $\eta = 0.1 \cdot E_N$.

Simulation results of the Šidák procedure regarding further simulation settings

Table S1: Impact of allocation bias on the mean FWERs and on $P_{RP}(\text{FWER} \leq 0.05)$ regarding different numbers of uncorrelated endpoints (m), RPs, sample sizes (N) and homogeneous allocation bias effects (η) by using the Šidák procedure.

RP	N	η	FWER [mean]		$P_{RP}(\text{FWER} \leq 0.05)$	
			m=2	m=5	m=2	m=5
CR	12	0.01795	0.0500	0.0500	0.60	0.60
		0.08975	0.0501	0.0501	0.60	0.60
		0.1795	0.0503	0.0504	0.61	0.61
	32	0.01024	0.0500	0.0500	0.55	0.55
		0.0512	0.0500	0.0501	0.55	0.55
		0.1024	0.0502	0.0502	0.55	0.55
	64	0.00711	0.0500	0.0500	0.54	0.54
		0.03555	0.0500	0.0500	0.54	0.54
		0.0711	0.0501	0.0501	0.54	0.54
BSD(3)	12	0.01795	0.0500	0.0500	0.50	0.50
		0.08975	0.0501	0.0501	0.50	0.50
		0.1795	0.0506	0.0507	0.52	0.52
	32	0.01024	0.0500	0.0500	0.34	0.34
		0.0512	0.0502	0.0502	0.34	0.34
		0.1024	0.0506	0.0508	0.34	0.34
	64	0.00711	0.0500	0.0500	0.15	0.15
		0.03555	0.0502	0.0503	0.15	0.15
		0.0711	0.0508	0.0510	0.15	0.15
MP(3)	12	0.01795	0.0500	0.0500	0.18	0.18
		0.08975	0.0508	0.0509	0.18	0.18
		0.1795	0.0531	0.0537	0.18	0.18
	32	0.01024	0.0500	0.0500	0.03	0.03
		0.0512	0.0507	0.0508	0.03	0.03
		0.1024	0.0527	0.0534	0.03	0.03
	64	0.00711	0.0500	0.0500	0.00	0.00
		0.03555	0.0507	0.0509	0.00	0.00
		0.0711	0.0527	0.0534	0.00	0.00

Table S1: Impact of allocation bias on the mean FWERs and on $P_{RP}(\text{FWER} \leq 0.05)$ regarding different numbers of uncorrelated endpoints (m), RPs, sample sizes (N) and homogeneous allocation bias effects (η) by using the Šidák procedure.

RP	N	η	FWER [mean]		$P_{RP}(\text{FWER} \leq 0.05)$	
			m=2	m=5	m=2	m=5
PBR(4)	12	0.01795	0.0501	0.0501	0.03	0.03
		0.08975	0.0516	0.0519	0.03	0.03
		0.1795	0.0562	0.0574	0.03	0.03
	32	0.01024	0.0501	0.0501	0.00	0.00
		0.0512	0.0518	0.0522	0.00	0.00
		0.1024	0.0572	0.0589	0.00	0.00
	64	0.00711	0.0501	0.0501	0.00	0.00
		0.03555	0.0519	0.0523	0.00	0.00
		0.0711	0.0575	0.0594	0.00	0.00
RAR	12	0.01795	0.0500	0.0500	0.24	0.24
		0.08975	0.0507	0.0503	0.24	0.24
		0.1795	0.0527	0.0510	0.24	0.24
	32	0.01024	0.0500	0.0500	0.18	0.18
		0.0512	0.0503	0.0501	0.18	0.18
		0.1024	0.0513	0.0505	0.18	0.18
	64	0.00711	0.0500	0.0500	0.17	0.17
		0.03555	0.0502	0.0501	0.17	0.17
		0.0711	0.0507	0.0503	0.17	0.18
EBC(0.67)	12	0.01795	0.0500	0.0500	0.29	0.29
		0.08975	0.0506	0.0507	0.29	0.29
		0.1795	0.0524	0.0529	0.31	0.31
	32	0.01024	0.0500	0.0500	0.11	0.11
		0.0512	0.0506	0.0508	0.11	0.11
		0.1024	0.0525	0.0532	0.11	0.11
	64	0.00711	0.0500	0.0500	0.03	0.03
		0.03555	0.0506	0.0508	0.03	0.03
		0.0711	0.0526	0.0533	0.03	0.03
CHEN(2,0.67)	12	0.01795	0.0500	0.0500	0.18	0.18
		0.08975	0.0508	0.0510	0.18	0.18
		0.1795	0.0532	0.0538	0.19	0.19
	32	0.01024	0.0500	0.0501	0.01	0.01
		0.0512	0.0510	0.0513	0.01	0.01
		0.1024	0.0541	0.0551	0.01	0.01
	64	0.00711	0.0500	0.0501	0.00	0.00
		0.03555	0.0511	0.0514	0.00	0.00
		0.0711	0.0545	0.0557	0.00	0.00

Table S2: Impact of allocation bias on the mean FWERs and on $P_{RP}(\text{FWER} \leq 0.05)$ regarding different number of correlated endpoints (m), RPs, sample sizes (N) and homogeneous allocation bias effects (η) by using the Šidák procedure. For the correlation structure, we assume compound symmetry. Thus the diagonal elements of the correlation matrix are 1 and the others $\rho = 0.5$.

RP	N	η	FWER [mean]		$P_{RP}(\text{FWER} \leq 0.05)$	
			m=2	m=5	m=2	m=5
CR	12	0.01795	0.0500	0.0500	0.60	0.60
		0.08975	0.0501	0.0500	0.60	0.60
		0.1795	0.0502	0.0501	0.61	0.61
	32	0.01024	0.0500	0.0500	0.55	0.55
		0.0512	0.0500	0.0500	0.55	0.55
		0.1024	0.0501	0.0501	0.55	0.55
	64	0.00711	0.0500	0.0500	0.54	0.54
		0.03555	0.0500	0.0500	0.54	0.54
		0.0711	0.0501	0.0500	0.54	0.54
BSD(3)	12	0.01795	0.0500	0.0500	0.50	0.50
		0.08975	0.0501	0.0501	0.50	0.50
		0.1795	0.0504	0.0502	0.52	0.52
	32	0.01024	0.0500	0.0500	0.34	0.34
		0.0512	0.0501	0.0501	0.34	0.34
		0.1024	0.0504	0.0503	0.34	0.34
	64	0.00711	0.0500	0.0500	0.15	0.15
		0.03555	0.0501	0.0501	0.15	0.15
		0.0711	0.0506	0.0503	0.15	0.155
MP(3)	12	0.01795	0.0500	0.0500	0.18	0.18
		0.08975	0.0505	0.0503	0.18	0.18
		0.1795	0.0521	0.0512	0.18	0.18
	32	0.01024	0.0500	0.0500	0.03	0.03
		0.0512	0.0505	0.0503	0.03	0.03
		0.1024	0.0518	0.0511	0.03	0.03
	64	0.00711	0.0500	0.0500	0.00	0.00
		0.03555	0.0505	0.0503	0.00	0.00
		0.0711	0.0518	0.0511	0.00	0.00
PBR(4)	12	0.01795	0.0500	0.0500	0.03	0.03
		0.08975	0.0510	0.0506	0.03	0.03
		0.1795	0.0542	0.0525	0.03	0.03
	32	0.01024	0.0500	0.0500	0.00	0.00
		0.0512	0.0512	0.0507	0.00	0.00
		0.1024	0.0548	0.0530	0.00	0.00
	64	0.00711	0.0500	0.0500	0.00	0.00
		0.03555	0.0512	0.0508	0.00	0.00
		0.0711	0.0550	0.0532	0.00	0.00

Table S2: Impact of allocation bias on the mean FWERs and on $P_{RP}(\text{FWER} \leq 0.05)$ regarding different number of correlated endpoints (m), RPs, sample sizes (N) and homogeneous allocation bias effects (η) by using the Šidák procedure. For the correlation structure, we assume compound symmetry. Thus the diagonal elements of the correlation matrix are 1 and the others $\rho = 0.5$.

RP	N	η	FWER [mean]		$P_{RP}(\text{FWER} \leq 0.05)$	
			m=2	m=5	m=2	m=5
RAR	12	0.01795	0.0500	0.0500	0.24	0.24
		0.08975	0.0505	0.0503	0.24	0.24
		0.1795	0.0518	0.0510	0.24	0.24
	32	0.01024	0.0500	0.0500	0.18	0.18
		0.0512	0.0502	0.0501	0.18	0.18
		0.1024	0.0509	0.0505	0.18	0.18
	64	0.00711	0.0500	0.0500	0.17	0.17
		0.03555	0.0501	0.0501	0.17	0.17
		0.0711	0.0505	0.0503	0.17	0.17
EBC(0.67)	12	0.01795	0.0500	0.0500	0.29	0.29
		0.08975	0.0504	0.0502	0.29	0.29
		0.1795	0.0516	0.0510	0.31	0.31
	32	0.01024	0.0500	0.0500	0.11	0.11
		0.0512	0.0504	0.0503	0.11	0.11
		0.1024	0.0517	0.0511	0.11	0.11
	64	0.00711	0.0500	0.0500	0.03	0.03
		0.03555	0.0504	0.0503	0.03	0.03
		0.0711	0.0518	0.0511	0.03	0.03
CHEN(2,0.67)	12	0.01795	0.0500	0.0500	0.18	0.18
		0.08975	0.0505	0.0503	0.18	0.18
		0.1795	0.0522	0.0513	0.19	0.19
	32	0.01024	0.0500	0.0500	0.01	0.01
		0.0512	0.0507	0.0504	0.01	0.01
		0.1024	0.0528	0.0517	0.01	0.01
	64	0.00711	0.0500	0.0500	0.00	0.00
		0.03555	0.0508	0.0505	0.00	0.00
		0.0711	0.0530	0.0519	0.00	0.00

Table S3: Impact of allocation bias on the mean FWERs and on $P_{RP}(\text{FWER} \leq 0.05)$ regarding different number of correlated endpoints (m), RPs, sample sizes (N) and homogeneous allocation bias effects (η) by using the Šidák procedure. For the correlation structure, we assume compound symmetry. Thus the diagonal elements of the correlation matrix are 1 and the others $\rho = 0.9$.

RP	N	η	FWER [mean]		$P_{RP}(\text{FWER} \leq 0.05)$	
			m=2	m=5	m=2	m=5
CR	12	0.01795	0.0500	0.0500	0.60	0.60
		0.08975	0.0500	0.0500	0.60	0.60
		0.1795	0.0502	0.0501	0.60	0.61
	32	0.01024	0.0500	0.0500	0.55	0.55
		0.0512	0.0500	0.0500	0.55	0.55
		0.1024	0.0501	0.0501	0.55	0.55
	64	0.00711	0.0500	0.0500	0.54	0.54
		0.03555	0.0500	0.0500	0.54	0.54
		0.0711	0.0501	0.0500	0.54	0.54
BSD(3)	12	0.01795	0.0500	0.0500	0.50	0.50
		0.08975	0.0501	0.0500	0.50	0.50
		0.1795	0.0503	0.0501	0.52	0.52
	32	0.01024	0.0500	0.0490	0.34	0.34
		0.0512	0.0501	0.0500	0.34	0.34
		0.1024	0.0503	0.0500	0.34	0.34
	64	0.00711	0.0500	0.0502	0.15	0.15
		0.03555	0.0501	0.0500	0.15	0.15
		0.0711	0.0504	0.0501	0.15	0.15
MP(3)	12	0.01795	0.0500	0.0500	0.18	0.18
		0.08975	0.0504	0.0502	0.18	0.18
		0.1795	0.0516	0.0508	0.18	0.18
	32	0.01024	0.0500	0.0500	0.03	0.03
		0.0512	0.0504	0.0502	0.03	0.03
		0.1024	0.0514	0.0507	0.03	0.03
	64	0.00711	0.0500	0.0500	0.00	0.00
		0.03555	0.0504	0.0502	0.00	0.00
		0.0711	0.0514	0.0507	0.00	0.00
PBR(4)	12	0.01795	0.0500	0.0500	0.03	0.03
		0.08975	0.0508	0.0504	0.03	0.03
		0.1795	0.0533	0.0516	0.03	0.03
	32	0.01024	0.0500	0.0500	0.00	0.00
		0.0512	0.0509	0.0505	0.00	0.00
		0.1024	0.0538	0.0519	0.00	0.00
	64	0.00711	0.0500	0.0500	0.00	0.00
		0.03555	0.0510	0.0505	0.00	0.00
		0.0711	0.0539	0.0521	0.00	0.00

Table S3: Impact of allocation bias on the mean FWERs and on $P_{RP}(\text{FWER} \leq 0.05)$ regarding different number of correlated endpoints (m), RPs, sample sizes (N) and homogeneous allocation bias effects (η) by using the Šidák procedure. For the correlation structure, we assume compound symmetry. Thus the diagonal elements of the correlation matrix are 1 and the others $\rho = 0.9$.

RP	N	η	FWER [mean]		$P_{RP}(\text{FWER} \leq 0.05)$	
			m=2	m=5	m=2	m=5
RAR	12	0.01795	0.0500	0.0500	0.24	0.24
		0.08975	0.0504	0.0502	0.24	0.24
		0.1795	0.0514	0.0507	0.24	0.24
	32	0.01024	0.0500	0.0500	0.18	0.18
		0.0512	0.0502	0.0501	0.18	0.18
		0.1024	0.0507	0.0504	0.18	0.18
	64	0.00711	0.0500	0.0500	0.17	0.17
		0.03555	0.0501	0.0500	0.17	0.17
		0.0711	0.0504	0.0502	0.17	0.17
EBC(0.67)	12	0.01795	0.0500	0.0500	0.29	0.29
		0.08975	0.0503	0.0502	0.29	0.29
		0.1795	0.0513	0.0506	0.31	0.31
	32	0.01024	0.0500	0.0500	0.11	0.11
		0.0512	0.0503	0.0502	0.11	0.11
		0.1024	0.0513	0.0507	0.11	0.11
	64	0.00711	0.0500	0.0500	0.03	0.03
		0.03555	0.0503	0.0502	0.03	0.03
		0.0711	0.0513	0.0507	0.03	0.03
CHEN(2,0.67)	12	0.01795	0.0500	0.0500	0.18	0.18
		0.08975	0.0504	0.0502	0.18	0.18
		0.1795	0.0517	0.0508	0.19	0.19
	32	0.01024	0.0500	0.0500	0.01	0.01
		0.0512	0.0505	0.0503	0.01	0.01
		0.1024	0.0522	0.0511	0.01	0.01
	64	0.00711	0.0500	0.0500	0.00	0.00
		0.03555	0.0506	0.0503	0.00	0.00
		0.0711	0.0524	0.0512	0.00	0.00

Simulation results regarding the Šidák procedure for heterogeneous allocation bias effects

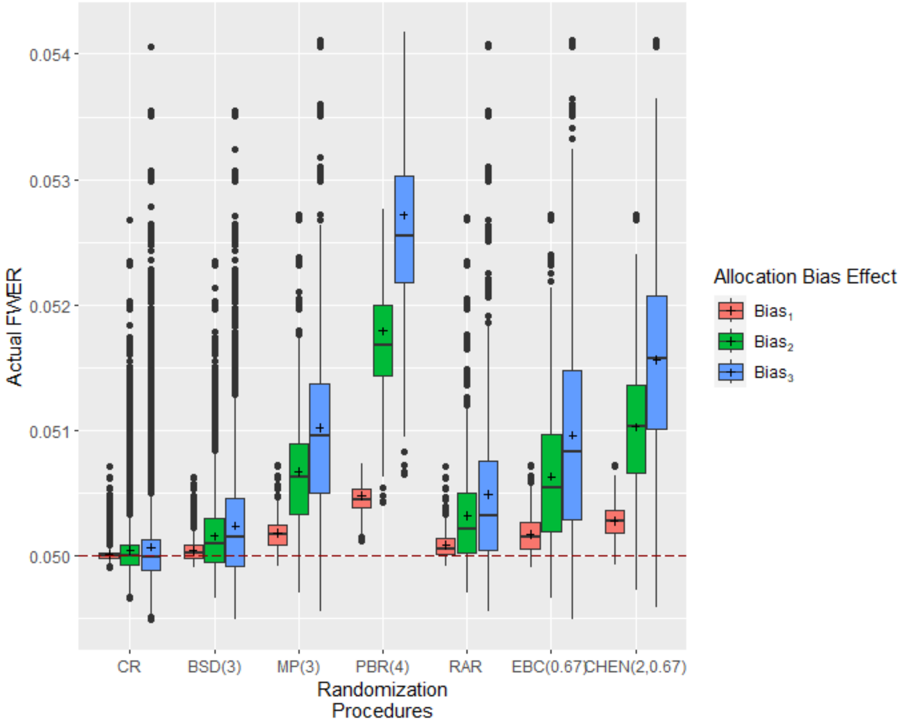

Figure S3: Distribution of the actual FWERs of the Šidák procedure for different endpoint-specific allocation bias effects. Simulations based on r=100 000 randomization lists, a sample size of $N = 32$ patients, $m = 5$ uncorrelated endpoints and allocation bias effects $Bias_1 = (0, 0, 0.01, 0.01, 0.05)^T \cdot E_{32} = (0, 0, 0.01024, 0.01024, 0.0512)^T$, $Bias_2 = (0, 0, 0, 0, 0.1)^T \cdot E_{32} = (0, 0, 0, 0, 0.1024)^T$ and $Bias_3 = (0.01, 0.01, 0.05, 0.05, 0.1)^T \cdot E_{32} = (0.01024, 0.01024, 0.0512, 0.0512, 0.1024)^T$.

Table S4: Mean FWERs and P_{RP}(FWER $\leq$ 0.05) of the Šidák procedure regarding a bias effect of $Bias_1 = (0, 0, 0.01, 0.01, 0.05)^T \cdot E_{32}$ in a clinical trial with $m = 5$ uncorrelated endpoints and $N = 32$ patients.

ρ	RPs	FWER [mean]	$P_{RP}(FWER \leq 0.05)$
	CR	0.0500	0.55
	BSD(3)	0.0500	0.34
	MP(3)	0.0502	0.03
0	PBR(4)	0.0505	0
	RAR	0.0501	0.18
	EBC(0.67)	0.0502	0.11
	CHEN(2,0.67)	0.0503	0.01

Table S5: Mean FWERs and P_{RP}(FWER $\leq$ 0.05) of the Šidák procedure regarding a bias effect of $Bias_2 = (0, 0, 0, 0, 0.1)^T \cdot E_{32}$ in a clinical trial with $m = 5$ uncorrelated endpoints and $N = 32$ patients.

ρ	RPs	FWER [mean]	$P_{RP}(FWER \leq 0.05)$
	CR	0.0500	0.55
	BSD(3)	0.0502	0.34
	MP(3)	0.0507	0.03
0	PBR(4)	0.0518	0
	RAR	0.0503	0.18
	EBC(0.67)	0.0506	0.11
	CHEN(2,0.67)	0.0510	0.01

Table S6: Mean FWERs and P_{RP}(FWER $\leq$ 0.05) of the Šidák procedure regarding a bias effect of $Bias_3 = (0.01, 0.01, 0.05, 0.05, 0.1)^T \cdot E_{32}$ in a clinical trial with $m = 5$ uncorrelated endpoints and $N = 32$ patients.

ρ	RPs	FWER [mean]	$P_{RP}(FWER \leq 0.05)$
	CR	0.0501	0.55
	BSD(3)	0.0502	0.34
	MP(3)	0.0510	0.03
0	PBR(4)	0.0527	0
	RAR	0.0505	0.18
	EBC(0.67)	0.0510	0.11
	CHEN(2,0.67)	0.0516	0.01

S2: Detailed simulation results regarding the impact of allocation bias by using the all-or-none procedure

In the following, we show detailed results of the simulation study introduced in Section 3.2. The simulation study analyzes the impact of allocation bias on the actual type I error (T1E) of the all-or-none procedure regarding different randomization procedures (RPs).

Boxplots of the simulation results regarding the all-or-none procedure presented in the manuscript

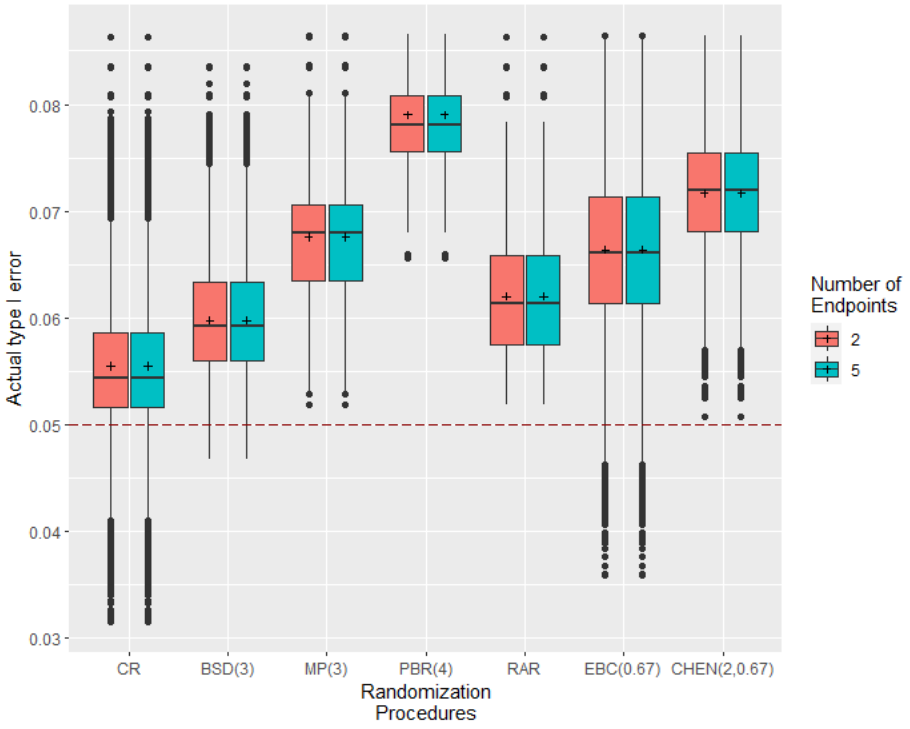

Figure S4: Distribution of the actual T1Es of the all-or-none procedure for different numbers of endpoints. Simulations based on r=100 000 randomization lists, a sample size of $N = 32$ patients and homogeneous allocation bias effects $\eta = 0.1 \cdot E_N = 0.1024$ for all endpoints.

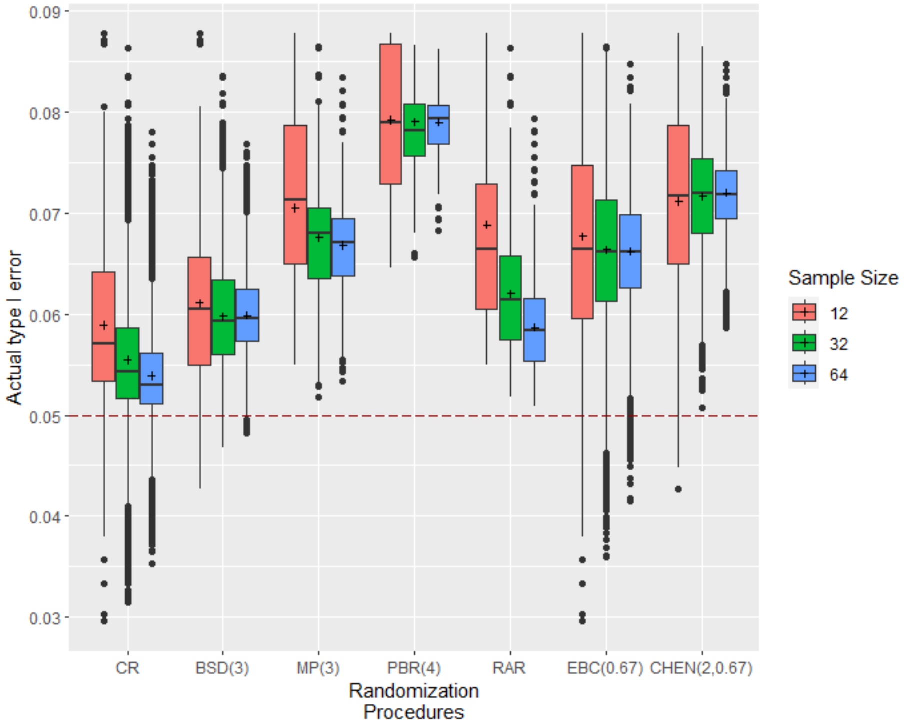

Figure S5: Distribution of the actual T1Es of the all-or-none procedure for different sample sizes. Simulations based on r=100 000 randomization lists, $m = 2$ endpoints and homogeneous allocation bias effects of $\eta = 0.1 \cdot E_N$.

Simulation results of the all-or-none procedure regarding further simulation settings

Table S7: Impact of allocation bias on the mean T1Es and on $P_{RP}(\text{T1E} \leq 0.05)$ regarding different numbers of endpoints (m), RPs, sample sizes (N) and homogeneous allocation bias effects η using the all-or-none procedure.

RP	N	η	T1E [mean]		$P_{RP}(\text{T1E} \leq 0.05)$	
			m=2	m=5	m=2	m=5
CR	12	0.01795	0.0509	0.0509	0.09	0.09
		0.08975	0.0544	0.0544	0.09	0.09
		0.1795	0.0589	0.0589	0.09	0.09
	32	0.01024	0.0505	0.0505	0.08	0.08
		0.0512	0.0527	0.0527	0.08	0.08
		0.1024	0.0555	0.0555	0.09	0.09
	64	0.00711	0.0504	0.0504	0.09	0.09
		0.03555	0.0519	0.0519	0.09	0.09
		0.0711	0.0539	0.0539	0.09	0.09
BSD(3)	12	0.01795	0.0511	0.0511	0.05	0.05
		0.08975	0.0555	0.0555	0.05	0.05
		0.1795	0.0611	0.0611	0.06	0.06
	32	0.01024	0.0509	0.0509	0.01	0.01
		0.0512	0.0548	0.0548	0.01	0.01
		0.1024	0.0598	0.0598	0.02	0.02
	64	0.00711	0.0509	0.0509	0.00	0.00
		0.03555	0.0548	0.0548	0.00	0.00
		0.0711	0.0599	0.0599	0.00	0.00
MP(3)	12	0.01795	0.0519	0.0519	0.00	0.00
		0.08975	0.0598	0.0598	0.00	0.00
		0.1795	0.0705	0.0705	0.00	0.00
	32	0.01024	0.0516	0.0516	0.00	0.00
		0.0512	0.0584	0.0584	0.00	0.00
		0.1024	0.0676	0.0676	0.00	0.00
	64	0.00711	0.0515	0.0515	0.00	0.00
		0.03555	0.0580	0.0580	0.00	0.00
		0.0711	0.0668	0.0668	0.00	0.00

Table S7: Impact of allocation bias on the mean T1Es and on $P_{RP}(\text{T1E} \leq 0.05)$ regarding different numbers of endpoints (m), RPs, sample sizes (N) and homogeneous allocation bias effects η using the all-or-none procedure.

RP	N	η	T1E [mean]		$P_{RP}(\text{T1E} \leq 0.05)$	
			m=2	m=5	m=2	m=5
PBR(4)	12	0.01795	0.0525	0.0525	0.00	0.00
		0.08975	0.0635	0.0635	0.00	0.00
		0.1795	0.0792	0.0792	0.00	0.00
	32	0.01024	0.0525	0.0525	0.00	0.00
		0.0512	0.0633	0.0633	0.00	0.00
		0.1024	0.0790	0.0790	0.00	0.00
	64	0.00711	0.0525	0.0525	0.00	0.00
		0.03555	0.0633	0.0633	0.00	0.00
		0.0711	0.0790	0.0790	0.00	0.00
RAR	12	0.01795	0.0517	0.0517	0.00	0.00
		0.08975	0.0590	0.0590	0.00	0.00
		0.1795	0.0688	0.0688	0.00	0.00
	32	0.01024	0.0511	0.0511	0.00	0.00
		0.0512	0.0558	0.0558	0.00	0.00
		0.1024	0.0620	0.0620	0.00	0.00
	64	0.00711	0.0508	0.0508	0.00	0.00
		0.03555	0.0542	0.0542	0.00	0.00
		0.0711	0.0587	0.0587	0.00	0.00
EBC(0.67)	12	0.01795	0.0516	0.0516	0.03	0.03
		0.08975	0.0585	0.0585	0.03	0.03
		0.1795	0.0677	0.0677	0.03	0.03
	32	0.01024	0.0515	0.0515	0.01	0.01
		0.0512	0.0578	0.0578	0.01	0.01
		0.1024	0.0664	0.0664	0.01	0.01
	64	0.00711	0.0515	0.0515	0.00	0.00
		0.03555	0.0577	0.0577	0.00	0.00
		0.0711	0.0662	0.0662	0.00	0.00
CHEN(2,0.67)	12	0.01795	0.0519	0.0519	0.00	0.00
		0.08975	0.0601	0.0601	0.00	0.00
		0.1795	0.0712	0.0712	0.00	0.00
	32	0.01024	0.0519	0.0519	0.00	0.00
		0.0512	0.0602	0.0602	0.00	0.00
		0.1024	0.0717	0.0717	0.00	0.00
	64	0.00711	0.0519	0.0519	0.00	0.00
		0.03555	0.0603	0.0603	0.00	0.00
		0.0711	0.0720	0.0720	0.00	0.00

Simulation results regarding the all-or-none procedure for heterogeneous allocation bias effects

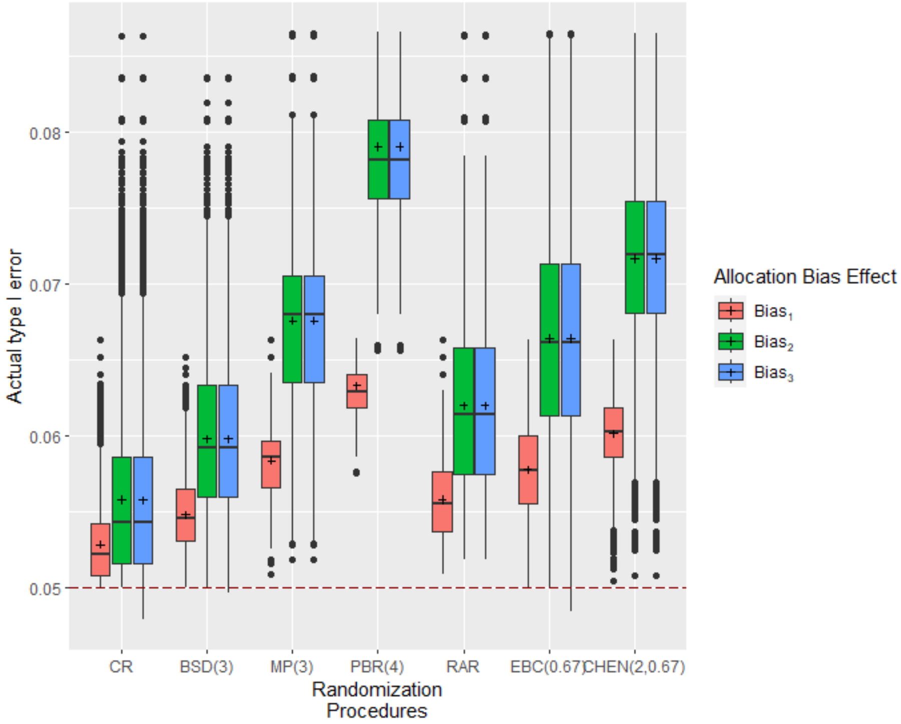

Figure S6: Distribution of the actual T1Es of the all-or-none procedure for different endpoint-specific allocation bias effects. Simulations based on r=100 000 randomization lists, a sample size of $N = 32$ patients, $m = 5$ uncorrelated endpoints and allocation bias effects $Bias_1 = (0, 0, 0.01, 0.01, 0.05)^T \cdot E_{32} = (0, 0, 0.01024, 0.01024, 0.0512)^T$, $Bias_2 = (0, 0, 0, 0, 0.1)^T \cdot E_{32} = (0, 0, 0, 0, 0.1024)^T$ and $Bias_3 = (0.01, 0.01, 0.05, 0.05, 0.1)^T \cdot E_{32} = (0.01024, 0.01024, 0.0512, 0.0512, 0.1024)^T$.

Table S8: Mean T1Es and $P_{RP}(\text{T1E} \leq 0.05)$ of the all-or-none procedure regarding a bias effect of $Bias_1 = (0, 0, 0.01, 0.01, 0.05)^T \cdot E_{32}$ in a clinical trial with $m = 5$ uncorrelated endpoints and $N = 32$ patients.

RPs	T1E [mean]	$P_{RP}(T1E \leq 0.05)$
CR	0.0558	0
BSD(3)	0.0598	0
MP(3)	0.0676	0
PBR(4)	0.0790	0
RAR	0.0620	0
EBC(0.67)	0.0664	0
CHEN(2,0.67)	0.0717	0

Table S9: Mean T1Es and $P_{RP}(\text{T1E} \leq 0.05)$ of the all-or-none procedure regarding a bias effect of $Bias_2 = (0, 0, 0, 0, 0.1)^T \cdot E_{32}$ in a clinical trial with $m = 5$ uncorrelated endpoints and $N = 32$ patients.

RPs	T1E [mean]	$P_{RP}(T1E \leq 0.05)$
CR	0.0529	0
BSD(3)	0.0548	0
MP(3)	0.0584	0
PBR(4)	0.0633	0
RAR	0.0558	0
EBC(0.67)	0.0578	0
CHEN(2,0.67)	0.0602	0

Table S10: Mean T1Es and $P_{RP}(\text{T1E} \leq 0.05)$ of the all-or-none procedure regarding a bias effect of $Bias_3 = (0.01, 0.01, 0.05, 0.05, 0.1)^T \cdot E_{32}$ in a clinical trial with $m = 5$ uncorrelated endpoints and $N = 32$ patients.

RPs	T1E [mean]	$P_{RP}(T1E \leq 0.05)$
CR	0.0558	0.08
BSD(3)	0.0598	0.01
MP(3)	0.0676	0
PBR(4)	0.0790	0
RAR	0.0620	0
EBC(0.67)	0.0664	0.01
CHEN(2,0.67)	0.0717	0

S3: Impact of allocation bias for the Šidák versus Bonferroni procedure

In the following, we present simulation results on the impact of bias in clinical trials evaluated by the Bonferroni or Šidák procedure. For the Šidák procedure, the correlation structure of the endpoints was considered using PCA, whereas for the Bonferroni procedure, potential correlation structures were ignored. The figures below demonstrate the differences and similarities in preventing allocation bias between these two approaches.

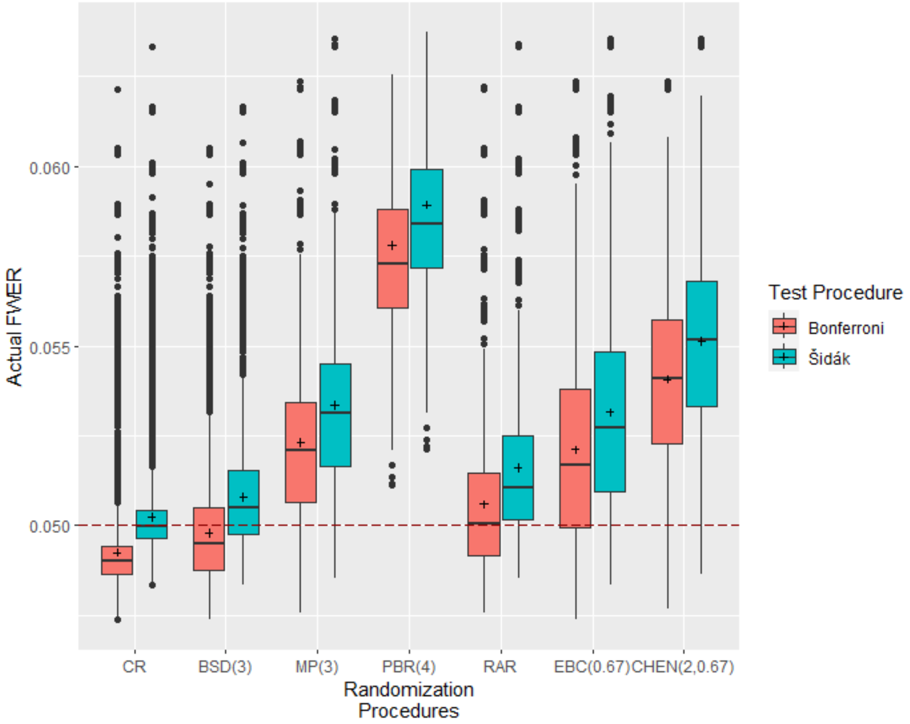

Figure S7: Distribution of the actual FWERs of the Bonferroni versus Šidák procedure under allocation bias. Simulations based on r=100 000 randomization lists, a sample size of $N = 32$ patients, $m = 5$ uncorrelated endpoints and allocation bias effects $\eta = (0.1, 0.1, 0.1, 0.1, 0.1)^T \cdot E_{32} = (0.1024, 0.1024, 0.1024, 0.1024, 0.1024)^T$.

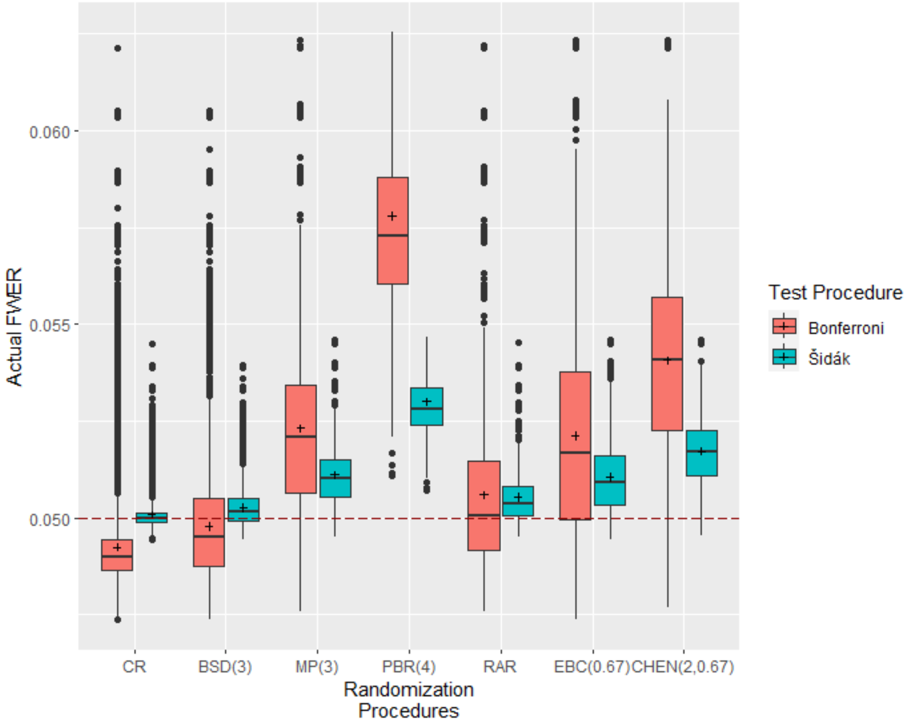

Figure S8: Distribution of the actual FWERs of the Bonferroni versus Šidák procedure under allocation bias. Simulations based on r=100 000 randomization lists, a sample size of $N = 32$ patients, $m = 5$ correlated endpoints and allocation bias effects $\eta = (0.1, 0.1, 0.1, 0.1, 0.1)^T \cdot E_{32}$. For the correlation structure, we assume compound symmetry. Thus, the diagonal elements of the correlation matrix are 1 and the others $\rho = 0.5$.

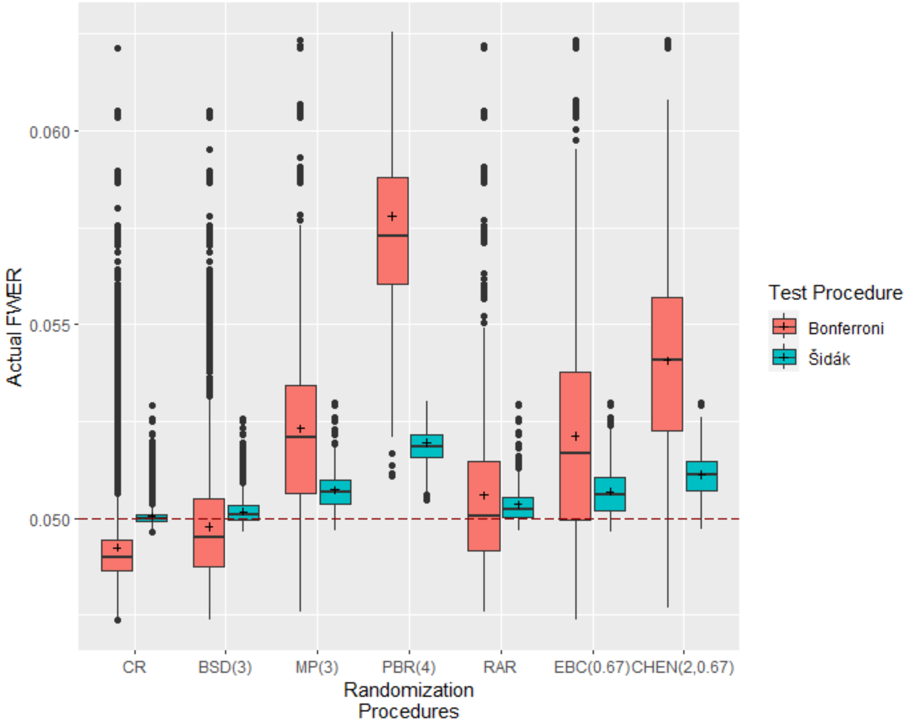

Figure S9: Distribution of the actual FWERs of the Bonferroni versus Šidák procedure under allocation bias. Simulations based on r=100 000 randomization lists, a sample size of $N = 32$ patients, $m = 5$ correlated endpoints and allocation bias effects $\eta = (0.1, 0.1, 0.1, 0.1, 0.1)^T \cdot E_{32}$. For the correlation structure, we assume compound symmetry. Thus, the diagonal elements of the correlation matrix are 1 and the others $\rho = 0.9$.
